# Oral health-related quality of life, experience and satisfaction in adolescents treated for dental crowding with self-ligating or conventional fixed appliances: a multicentre randomized controlled trial

**DOI:** 10.1093/ejo/cjag040

**Published:** 2026-06-08

**Authors:** Linda Bokander Matilainen, Kristina Johansson, Sofia Petrén, Anna Brechter, Michal Wiaderny, Ambrosina Michelotti, Liselotte Paulsson

**Affiliations:** Department of Orthodontics, Faculty of Odontology, Malmö University, Carl Gustavs väg 34, SE-205 06 Malmö, Sweden; Department of Orthodontics, Faculty of Odontology, Malmö University, Carl Gustavs väg 34, SE-205 06 Malmö, Sweden; Department of Orthodontics, Östersund Hospital, SE-831 83 Östersund, Sweden; Department of Orthodontics, Faculty of Odontology, Malmö University, Carl Gustavs väg 34, SE-205 06 Malmö, Sweden; Bernhold Ortodonti, Private Orthodontic Practice, Ekslingan 10, SE-254 67 Helsingborg, Sweden; Tandreglering Falun, Public Dental Service Dalarna, Box 335, SE-791 27 Falun, Sweden; Section of Orthodontics, Department of Neurosciences, Reproductive Sciences and Oral Sciences, University of Naples Federico II, Via Sergio Pansini 5, I-80131 Naples, Italy; Department of Orthodontics, Faculty of Odontology, Malmö University, Carl Gustavs väg 34, SE-205 06 Malmö, Sweden

**Keywords:** dental patient reported outcome, fixed appliance treatment, orthodontic brackets, self-ligating bracket, conventional bracket, oral health-related quality of life, CPQ 11-14, patient satisfaction

## Abstract

**Background:**

Dental crowding affects nearly half of European adolescents and can negatively impact oral health-related quality of life (OHRQoL), particularly emotional and social well-being dimensions. Orthodontic treatment aims to improve function, aesthetics, and psychosocial well-being, yet active treatment may temporarily impair OHRQoL due to pain, dietary restrictions, and oral hygiene challenges. Understanding how conditions affect patients requires both objective and subjective assessments, yet patient reported outcomes remain underreported in orthodontic research, especially when comparing treatment techniques.

**Objectives:**

To evaluate and compare OHRQoL, treatment motivation, expectations, quality of care and attention, and satisfaction in adolescents with dental crowding treated with two fixed appliance systems without extractions, and to explore factors associated with satisfaction with treatment outcomes.

**Trial design:**

Multicentre, two-arm, parallel-group, superiority randomized controlled trial, with pooled analyses of both treatment groups, sex-stratified comparisons, and analyses exploring associations.

**Methods:**

Adolescents (*n* = 132), 12–17 years, with dental crowding were randomized with 1:1 ratio using computer generated random permuted blocks, stratified by sex, to receive fixed appliance treatment without extractions with either a conventional bracket system (CB; Victory) or a passive self-ligating bracket system (PSLB; DamonQ) at four orthodontic clinics. Primary outcome was Child Perceptions Questionnaire 11-14 (CPQ) score change. Two items were omitted; sensitivity analysis confirmed minimal impact on results. Secondary outcomes were patient experience and satisfaction questionnaires (10-point scale) assessed before treatment (T0), post-alignment (T1), and post-treatment (T2). Because of the nature of the treatments, only assessors could be blinded.

**Results:**

Both the CB (♀39, ♂31) and PSLB (♀34, ♂28) groups reported improved OHRQoL (median CPQ score change T0-T2 for total sample: 4.5; *r* = 0.37, *P* < .001) with minor intergroup differences. Oral symptoms and functional limitations worsened at T1 then improved by T2 (median change T0–T2 for each domain: 1, *r* = 0.23, 0.27, respectively, *P* < .001), whereas emotional and social well-being improved throughout treatment (median change T0–T2 for each domain: 1; *r* = 0.28, *P* < .001). Median scores were high for motivation (9) and overall satisfaction with treatment outcomes (≥9), and low for regretting having started treatment (1). Correlations between treatment expectations and satisfaction were observed (*ρ* = 0.330, *P* < .001). Minor harms expected during orthodontic treatment included plaque accumulation, chafing and gingivitis.

**Conclusions:**

This study indicates that fixed appliance treatment for dental crowding leads to improvements in OHRQoL, with temporary declines during treatment, and high patient satisfaction in the studied sample. Patients reported satisfaction with the changes achieved, dental appearance, and did not regret having started treatment. The results demonstrate possible benefits of treatment, patient-centred care, and support the use of patient reported outcomes in orthodontic practice.

**Trial registration:**

ClinicalTrials.gov (NCT05664282) 2022-11-24.

## Introduction

Dental crowding affects 42%–51% of European youth and is a primary reason for orthodontic treatment [1–3]. Crowding can negatively impact the emotional and social well-being dimensions of oral health-related quality of life (OHRQoL), which has been associated with bullying, low self-esteem, and high psychological distress among adolescents [4, 5]. Fixed appliance treatment aims to improve function, aesthetics, and provide psychosocial benefits to enhance patients’ quality of life (QoL). To understand a condition’s impact on a patient, both objective and subjective aspects must be considered, as the clinical severity of a condition may not align with patients’ personal perception [6].

Dental patient reported outcomes (dPROs) are patients’ own reports [6], and are essential for treatment planning, communication, and resource allocation. However, dPROs remain underreported in orthodontic research, only one-fourth of randomized controlled trials (RCTs) and about one-sixth of observational studies in the five leading orthodontic journals reflect patient perspectives [7, 8].

OHRQoL emerged from evidence linking oral conditions to overall health and QoL [9]. It is a multidimensional concept without any strict definition [10]. Locker described it as ‘the impact of oral disorders on aspects of everyday life that are important to patients and persons, with those impacts being of sufficient magnitude, whether in terms of severity, frequency, or duration, to affect an individual’s perception of their life overall’ [11]. Due to developmental differences in cognition, social maturity, and reading ability, various dPRO measures (dPROMs) have been developed such as the Child Perceptions Questionnaire 11-14 (CPQ11-14) and the Psychosocial Impact of Dental Aesthetics Questionnaire (PIDAQ). However, measurement diversity complicates comparison and synthesis of evidence [7]. While the PIDAQ, developed for orthodontic patients, assesses psychosocial effects of dental appearance, it does not evaluate function [12]. The CPQ11-14 [13] is a generic, reliable, and validated dPROM previously used in orthodontic RCTs and observational studies [7, 8].

Active orthodontic treatment can worsen OHRQoL, particularly in the early phases of treatment, e.g., due to pain, dietary restrictions, and oral hygiene challenges [14, 15]. Following treatment, OHRQoL generally improves [15]. Adolescents with passive self-ligating brackets (PSLB) have reported less pain and analgesic use than those with conventional brackets (CB) [16]. Meanwhile, a South American study in adults reported poorer OHRQoL with PSLB at 6 months of treatment, whereas Asian studies in adolescents and young adults reported no difference between systems [17–20].

Research on OHRQoL, particularly orthodontic RCTs, remains limited [21, 22]. Many existing studies focus on specific phases of treatment [17, 20, 23, 24]. In addition, cultural factors may influence both perceptions of malocclusions and interpretation of OHRQoL [25, 26]. Therefore, longitudinal studies comparing different treatment techniques across diverse populations are needed.

Furthermore, patient satisfaction is a dPRO associated with quality of patient-orthodontist relationship [27], pain and discomfort, motivation, care and attention [28]. Children and adolescents may seek orthodontic care for aesthetic and social reasons and may be influenced by peers and media [5, 29]. Therefore, it is important to explore adolescents’ expectations to ensure that they are realistic and achievable. Feldmann *et al*. developed dPROMs to assess adolescents’ expectations, motivation, quality of care and attention, pain and discomfort, and satisfaction with orthodontic treatment [28, 30]. Meanwhile, a Cochrane review on children and adolescents with dental crowding found that none of the included RCTs assessed patient satisfaction [2], reflecting a clinician-centred focus in research [31].

Therefore, the overall objective of this study was to assess and compare dPROs in adolescents (12–17 years) with dental crowding, treated without extractions using either a PSLB or a CB system, in a multicentre RCT. The study comprised three specific aims:

Primary aim to:

1. Compare OHRQoL between treatment groups, between boys and girls, and within the total sample before, during and after treatment.

Secondary aims to:

1. Compare treatment motivation, expectations, quality of care and attention, pain and discomfort, and satisfaction with treatment outcomes between the treatment groups and within the total sample.
2. Explore associations between patient satisfaction and demographics, clinical characteristics, objective treatment outcomes, OHRQoL, motivation, expectations, quality of care and attention, and pain and discomfort.

## Subjects and methods

### Trial design and ethics

This multicentre, two-arm parallel-group, superiority RCT also included pooled analyses of both treatment groups, sex-stratified comparisons, and exploratory analyses of associations in the total sample. It is part of the research project Crowded Displaced Teeth (CROWDIT), assessing multiple orthodontic outcomes. The trial protocol was approved by the Regional Ethical Review Board (Dnr:2014/647), in accordance with the Declaration of Helsinki, and registered on ClinicalTrials.gov (NCT05664282). Reporting adheres to the CONSORT guidelines [32]. No changes were made after trial commencement.

### Participants, settings, and eligibility criteria

Eligible patients awaiting approved subsidized orthodontic treatment were recruited at four clinics in Sweden (two public, one private, and one university clinic) and were treated by four orthodontic specialists.

#### Inclusion criteria

- 12–17 years of age at treatment initiation.
- Crowded and displaced anterior teeth in one or both arches.
- Sagittal relations within ± one cusp deviation from a normal sagittal relation.
- Overbite ≥ 0 mm.
- Normal transverse relation, or minor transverse dental discrepancy.
- Treatment needs 3, 4, or 5 according to the dental health component of the Index of Orthodontic Treatment Need Dental Health Component (IOTN DHC) [33].

#### Exclusion criteria

Treatment plan including extractions or surgical procedures, need for auxiliary appliances such as a transpalatal bar or Quad Helix; rheumatoid arthritis; missing permanent teeth; impacted teeth; previous orthodontic treatment; ongoing sucking habits; previous trauma to teeth or jaws with subjective, clinical, or radiographic findings; periapical pathology; probing depth of ≥5 mm at central incisors or first molars with a calibrated probe, screened at ≥4 sites per tooth; visible plaque Grade 3 [34]; or communication difficulties.

#### Enrolment

Eligible patients received verbal and written information and were consecutively invited to participate. Following assent and signed consent, an independent staff member randomized patients by opening the next sealed, opaque envelope in sequence. Treatment then followed the treatment protocol.

### Randomization, allocation and procedures

The allocation sequence to CB (Victory APCplus™) or PSLB (Damon Q™) was computer-generated in a 1:1 ratio using random permuted blocks of 10, stratified by sex. Sealed, sequentially numbered envelopes labelled ‘girl’ or ‘boy’ were prepared for each clinic. Baseline analysis and treatment protocol have been described previously [35].

### Questionnaires

All questionnaires were administered in paper format and completed independently at the clinic before scheduled treatment, without caregiver assistance. Staff provided clarification if needed. Completion took ∼10 min, but no time limit was set.

### Outcomes

Primary outcome was used to assess and compare OHRQoL between treatment groups, sexes, and the total sample:

- Modified Swedish CPQ11-14 [36] score at baseline (T0), score change during the alignment phase (T0–T1) and total treatment (T0–T2).

Secondary outcomes were used to compare motivation, expectations, experiences, and satisfaction with treatment outcomes between treatment groups, sexes and within the total sample, and to explore factors associated with treatment satisfaction:

- Modified Feldmann’s questionnaires: motivation and expectations quality of care and attention, pain and discomfort, and satisfaction with treatment outcome [28, 30] (T0, T1, and T2).
- Demographics and clinical characteristics: age, sex, Little’s irregularity index (LII) [37], IOTN DHC [33] (T0), weighted Peer Assessment Rating (wPAR) [38], and self-reported headache.
- Treatment effects: LII improvement T0–T2, wPAR percentage improvement T0–T2, treatment time T0–T2 (months).

#### OHRQoL assessment

OHRQoL was measured using a Swedish modified CPQ11-14 [13,36] comprising 37 items across four domains: oral symptoms (OS), functional limitations (FL), emotional well-being (EW), and social well-being (SW). Items were rated on a five-point Likert type scale: ‘never’ (0), ‘once or twice’ (1), ‘sometimes’ (2), ‘often’ (3), and ‘every day or almost every day’ (4), with higher scores indicating poorer OHRQoL. A ‘not applicable’ option was included and imputed with the median value for that item [36].

#### Motivation, expectations, quality of care and attention, pain and discomfort, and treatment satisfaction

These questionnaires were based on the validated Feldmann questionnaires [28, 30], scored on a ten-box end-anchored scale. A pilot with ten adolescents not involved in the study led to minor clarifications.

#### Missing

Missing questionnaires were treated as lost to follow-up. Missing item responses were imputed using the item median score. The impact of imputations was evaluated in a sensitivity analysis using complete questionnaires.

#### Sample size calculation

The CROWDIT research project included several sample size calculations across multiple outcomes; the final sample size was determined by the outcome requiring the largest sample. No prior research on the described population and instrument was available to inform sample size calculations for the OHRQoL outcome. Enrolment was halted at *n* = 132 due to COVID-19 and concurrent changes to regulations governing free orthodontic care, which affected two participating clinics. All CROWDIT participants were included in the present study, and these results may inform future sample size estimates.

### Blinding

Blinding of clinicians and patients was not feasible. Standardized protocols were applied for all participants, and assessments were performed on coded materials by independent, blinded observers who were not involved in treatment.

### Statistical analysis

Statistical analysis was advised by a statistician and performed using SPSS (version 30.0.0.0(172), IL, USA). Previously published intra- and interobserver reliability for baseline and treatment outcome assessments used the intraclass correlation coefficient (two-way mixed-effects model) [35]. Due to ordinal variables and non-normal distributions, intergroup comparisons used the Mann–Whitney *U* test, and intragroup analyses used the Wilcoxon signed-ranks test. Exploratory analysis of correlations was conducted using Spearman rank correlation, following Feldmann et al. [28]. Statistical significance was set at *P* < .05, with Bonferroni-adjusted thresholds (*P* < .0011–.0015) applied to exploratory analyses to account for multiple testing.

The CPQ11-14 version included 35 items; the last two items were omitted due to a printing error that affected the total and SW domain scores. No score adjustment was performed. Sensitivity analyses used the 16-item short-form regression version (RSF16), which is strongly correlated with the full version [39].

### Harms

Patients were followed as customary throughout orthodontic treatment, and adverse effects were reported to the principal researcher. Reasons for discontinuing treatment were the same as those for nonstudy participants.

## Results

**Figure 1:**
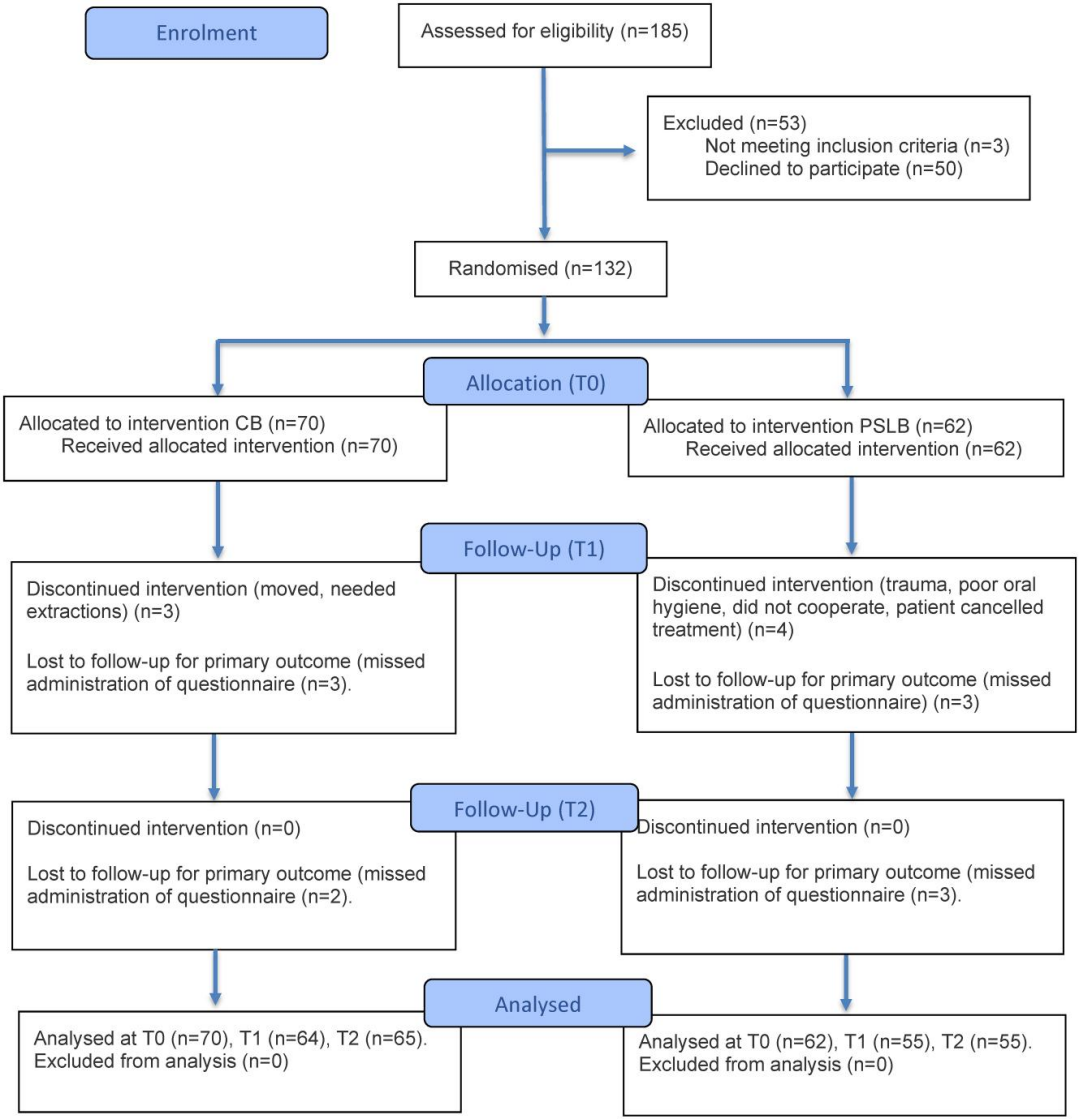
Adapted CONSORT 2025 flow diagram from Hopewell et al. [40].

**Figure 2:**
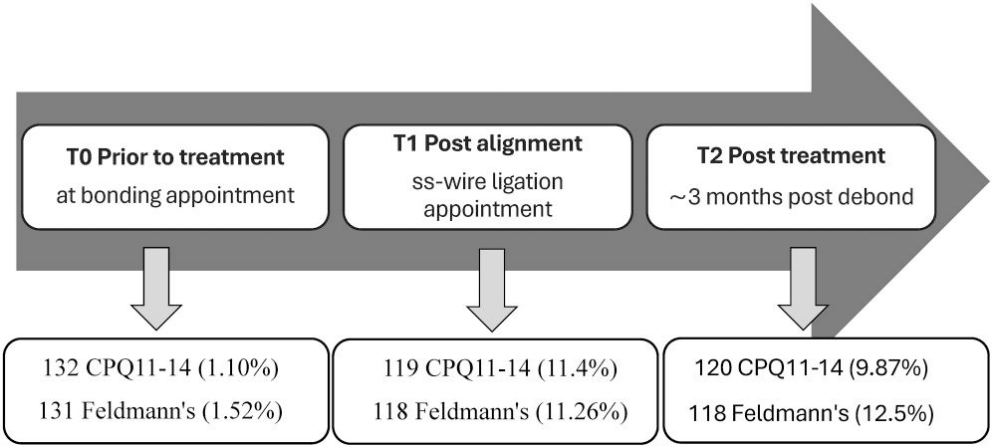
Number of questionnaires collected (percentage of missing items in parentheses).

Recruitment occurred between 2016 and 2020, and included 132 patients (Fig. 1, Table 1). The number of collected questionnaires and missing items at T0, T1, and T2 is shown in Fig. 2; missing data were assumed to be missing at random. The reasons for missing questionnaires are presented in Supplementary Table S1.

**Table 1:** Demographics, clinical characteristics, and treatment effects for the total sample, treatment group and sexes, and intergroup comparisons.

	Total sample								Groups									
Demographics and clinical characteristics						Percentiles			CB ♀39 ♂31		PSLB ♀34 ♂28		♀♂ NS^c^	BOY ♂59		GIRL ♀73		
Total sample	*n*	Mean	SD	Min	Max	25	50	75	*n*	Mean	*n*	Mean	*P*	*n*	Mean	*n*	Mean	*P*
Age	132	14.67	1.46	10.91	18.06	13.44	14.81	15.47	70	14.61	62	14.73	NS^a^	59	14.93	73	14.45	NS^a^
LII upper arch T0	127	9.74	3.50	1.09	23.10	7.35	9.33	11.51	68	10.13	59	9.29	NS^a^	54	9.54	68	10.06	NS^a^
LII lower arch T0	131	6.68	2.83	1.09	15.21	4.52	6.41	8.74	70	7.00	61	6.30	NS^a^	55	6.87	69	6.62	NS^a^
wPAR, T0	132	29.44	9.61	8.00	49.00	22.00	29.00	36.75	70	30.31	62	28.45	NS^b^	59	29.19	73	29.64	NS^b^
IOTN DHC	132	3.77	0.44	3	5	4	4	4	70	3.80	62	3.73	NS^b^	59	3.86	73	3.68	**.023^b^**
Headache reported at T0	12								8		4		NS^c^	4		8		NS^c^
Headache reported at T1	15								9		6		NS^c^	9		6		NS^c^
Headache reported at T2	8								4		4		NS^c^	2		6		NS^c^
Treatment effects																		
Time to alignment in months	120	12.82	5.30	3.84	29.73	8.94	11.66	15.57	65	12.12	55	13.63	NS^a^	56	13.81	64	11.95	NS^a^
Treatment time in months	124	22.94	7.44	10.32	42.55	17.00	21.60	27.36	67	21.74	57	24.35	NS^a^	56	22.97	68	22.92	NS^a^
LII upper arch T2	124	1.06	0.51	0.00	2.72	0.74	0.98	1.40	66	1.02	58	1.11	NS^a^	56	1.11	68	1.02	NS^a^
LII lower arch T2	124	1.10	0.61	0.00	2.99	0.71	1.06	1.44	66	1.10	58	1.11	NS^a^	56	1.16	68	1.06	NS^a^
LII reduction upper arch	121	−8.75	3.41	−21.78	−1.93	−10.32	−8.25	−6.59	64	−9.02	57	−8.44	NS^a^	54	−8.41	67	−9.02	NS^a^
LII reduction lower arch	123	−5.65	2.82	−13.46	−0.08	−7.17	−5.36	−3.71	66	−5.95	57	−5.29	NS^a^	55	−5.74	68	−5.57	NS^a^
wPAR at T2	132	6.29	6.61	0.00	40.00	2.00	4.00	8.00	70	6.24	62	6.34	NS^b^	59	5.39	73	7.01	NS^b^
wPAR improvement %	132	0.76	0.24	0.00	1.00	0.69	0.86	0.93	70	0.78	62	0.74	NS^b^	59	0.79	73	0.74	NS^b^

Statistical significance was defined as *P* < .05, *P*-values in bold are statistically significant.

*n*, number of cases; SD, standard deviation; Min, minimum; Max, maximum; CB, conventional brackets; PSLB, passive self-ligating brackets; *P*, *P*-value; LII, Little’s irregularity index; wPAR, weighted Peer Assessment Rating; IOTN DHC, Index of Orthodontic Treatment Need Dental Health Component; NS, non-significant; T0, baseline; T1, post alignment; T2, post treatment.

^a^Independent samples *t* test.

^b^Mann–Whitney *U* test.

^c^ *χ* ^2^.

### Oral health-related quality of life—aim 1

**Figure 3:**
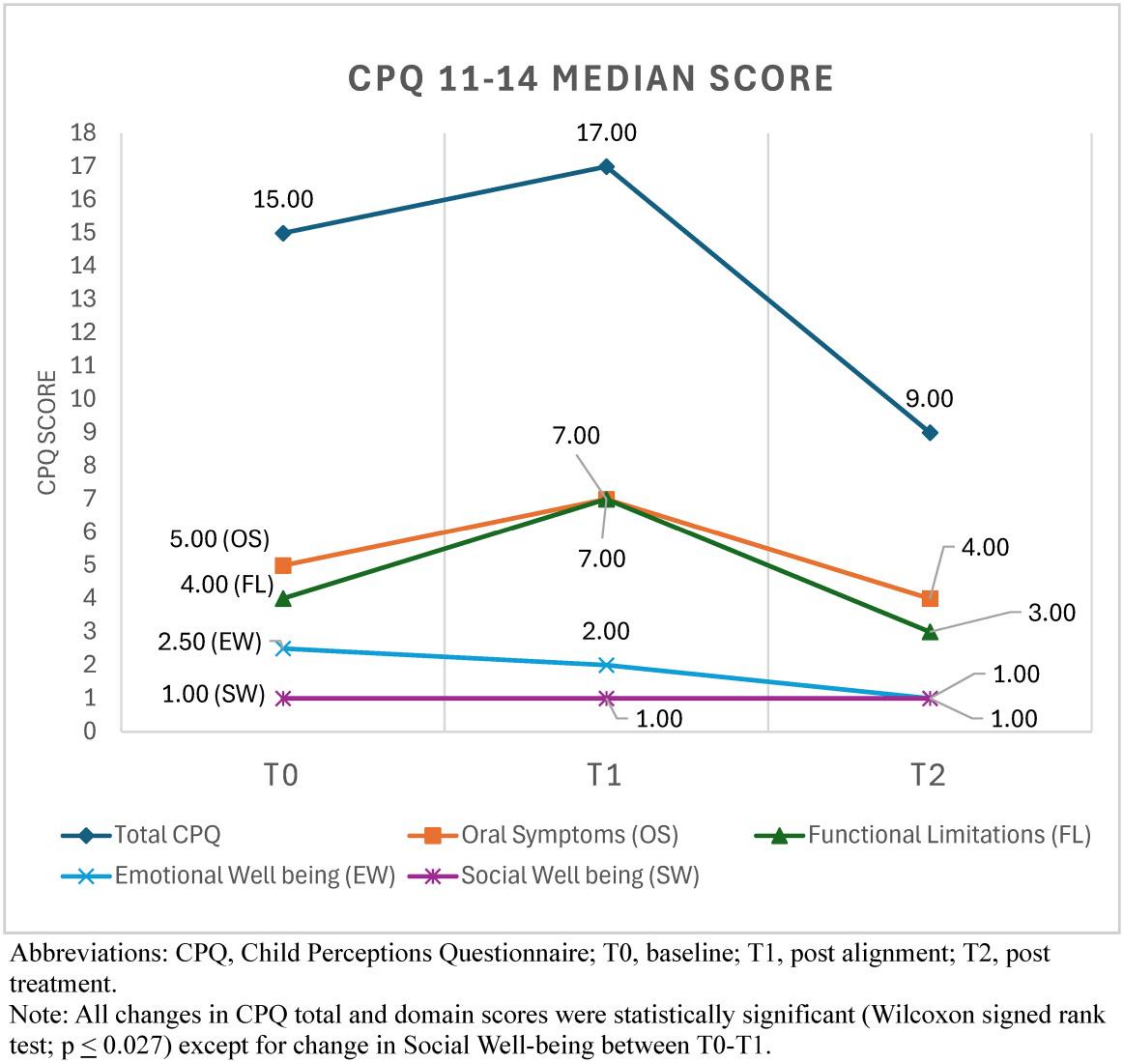
CPQ11-14 total and domain scores at T0, T1, and T2 presented as median scores.

OHRQoL for the total sample worsened at T1 compared with T0, then improved beyond baseline at T2 (*P* ≤ .013). The OS and FL domains followed this pattern, whereas EW and SW improved throughout treatment (Fig. 3; Supplementary Table S2). Changes in domain scores were statistically significant (*P* ≤ .027) except for SW from T0 to T1 (*P* = .743) (Supplementary Table S2). Statistically significant findings were supported by RSF16 sensitivity analysis (Supplementary Table S2).

Within the treatment groups, OHRQoL variation patterns were similar to those observed in the total sample. The CB group reported higher baseline SW scores and greater OS improvement, with small effect sizes (Table 2). Statistically significant inter- and intragroup findings were supported when analysed as RSF16, except for EW change T0–T1 within the PSLB group (Table 2, Supplementary Table S2).

**Table 2:** Intergroup comparison of CPQ11-14 scores between treatment groups and between boys and girls, using Mann–Whitney *U* test.

	CB				PSLB				Mann–Whitney *U*		
Variable	*n*	Mean	SD	Median	*n*	Mean	SD	Median	*U*	*r*	*P*-value
CPQ T0	70	18.51	10.65	17.00	62	16.97	13.64	13.00	1811.50	0.14	.102
CPQ T1	64	21.34	12.55	18.50	55	18.84	10.74	16.00	1588.50	0.08	.362
CPQ T2	65	11.08	7.27	8.00	55	12.07	9.59	10.00	1696.50	0.04	.633
OS T0	70	5.86	2.81	5.00	62	5.19	3.12	5.00	1792.50	0.15	.083
OS T1	64	7.23	3.02	7.00	55	7.00	2.94	7.00	1755.00	0.00	.980
OS T2	65	4.15	2.08	4.00	55	4.75	2.52	4.00	1523.00	0.13	.160
FL T0	70	4.93	3.38	4.00	62	4.48	3.42	4.00	1989.50	0.07	.410
FL T1	64	7.31	3.96	7.00	55	6.13	3.83	5.00	1451.50	0.15	.099
FL T2	65	3.40	2.65	3.00	55	2.87	2.39	2.00	1585.50	0.10	.284
EW T0	70	4.64	4.80	3.00	62	4.90	6.81	2.00	1930.50	0.10	.270
EW T1	64	4.06	5.73	2.00	55	3.31	4.31	2.00	1721.50	0.02	.836
EW T2	65	2.02	3.45	1.00	55	3.04	4.71	1.00	1576.50	0.11	.245
SW T0	70	3.09	3.62	2.00	62	2.39	4.09	1.00	1656.50	0.21	.**016**^a^
SW T1	64	2.73	3.74	1.00	55	2.40	3.24	1.00	1716.00	0.02	.812
SW T2	65	1.51	2.19	1.00	55	1.42	2.61	1.00	1606.00	0.09	.316
CPQ T0–T1	64	−3.30	12.14	−2.50	55	−1.71	13.84	−2.00	1695.50	0.03	.733
CPQ T0–T2	65	7.25	9.82	6.00	55	4.60	15.40	3.00	1561.00	0.11	.234
OS T0–T1	64	−1.58	2.82	−1.50	55	−1.82	3.28	−2.00	1648.00	0.06	.551
OS T0–T2	65	1.55	2.60	1.00	55	0.27	2.89	0.00	1262.50	0.25	.**005**^b^
FL T0–T1	64	−2.50	4.66	−2.00	55	−2.00	4.38	−1.00	1577.50	0.09	.331
FL T0–T2	65	1.58	3.78	2.00	55	1.27	3.73	1.00	1730.50	0.03	.765
EW T0–T1	64	0.50	5.03	1.00	55	1.96	6.90	0.00	1677.50	0.04	.661
EW T0–T2	65	2.58	4.79	1.00	55	1.95	7.89	1.00	1689.50	0.05	.606
SW T0–T1	64	0.28	4.17	0.00	55	0.15	5.22	−1.00	1530.50	0.11	.219
SW T0–T2	65	1.52	2.73	1.00	55	1.11	4.92	0.00	1526.50	0.13	.164

	BOY				GIRL				Mann–Whitney *U*		
Variable	*n*	Mean	SD	Median	*n*	Mean	SD	Median	*U*	*r*	*P*-value
Total T0	59	15.76	11.50	12.00	73	19.42	12.44	18.00	1692.50	0.18	.**035**^c^
Total T1	54	18.76	11.57	14.00	65	21.37	11.88	19.00	1470.50	0.14	.129
Total T2	56	11.55	9.08	8.50	64	11.52	7.82	9.00	1763.00	0.01	.880
OS T0	59	5.56	3.16	5.00	73	5.53	2.82	5.00	2093.50	0.02	.784
OS T1	54	7.43	3.09	7.00	65	6.88	2.88	7.00	1623.00	0.07	.480
OS T2	56	4.46	2.50	4.00	64	4.39	2.13	4.00	1762.50	0.01	.877
FL T0	59	4.93	3.60	4.00	73	4.55	3.24	4.00	2054.00	0.04	.649
FL T1	54	6.19	4.22	5.00	65	7.25	3.64	7.00	1418.50	0.17	.072
FL T2	56	3.27	2.53	3.00	64	3.06	2.56	3.00	1718.50	0.04	.698
EW T0	59	3.02	4.59	1.00	73	6.18	6.32	4.00	1404.50	0.30	**<**.**001**^d^
EW T1	54	2.89	4.64	1.00	65	4.40	5.43	2.00	1408.00	0.17	.060
EW T2	56	2.14	3.88	1.00	64	2.78	4.27	1.00	1678.00	0.06	.531
SW T0	59	2.25	3.37	1.00	73	3.16	4.17	2.00	1768.00	0.16	.071
SW T1	54	2.26	2.93	1.00	65	2.85	3.93	1.00	1582.50	0.09	.348
SW T2	56	1.68	3.07	1.00	64	1.28	1.56	1.00	1736.50	0.03	.761
CPQ T0–T1	54	−3.02	11.92	−2.00	65	−2.18	13.77	−2.00	1696.00	0.03	.755
CPQ T0–T2	56	4.04	13.47	2.50	64	7.78	11.81	6.00	1442.50	0.17	.066
OS T0–T1	54	−1.81	3.29	−2.00	65	−1.58	2.83	−2.00	1728.50	0.01	.888
OS T0–T2	56	1.11	3.18	1.00	64	0.84	2.44	0.50	1757.50	0.02	.856
FL T0–T1	54	−1.43	4.77	−1.00	65	−2.97	4.21	−2.00	1403.00	0.17	.059
FL T0–T2	56	1.54	3.75	1.00	64	1.36	3.77	1.50	1729.00	0.03	.741
EW T0–T1	54	0.17	5.05	0.00	65	2.02	6.58	2.00	1314.50	0.22	.**018**^e^
EW T0–T2	56	0.82	5.88	0.00	64	3.58	6.56	3.00	1308.50	0.23	.**010**^f^
SW T0–T1	54	0.06	4.17	0.00	65	0.35	5.06	0.00	1673.00	0.04	.661
SW T0–T2	56	0.57	3.71	1.00	64	2.00	3.92	1.00	1448.50	0.17	.067

Statistical significance was defined as *P* < .05. *P*-values in bold are statistically significant. Statistically significant findings were supported by sensitivity analyses using the CPQ11-14 16-item short-form regression version (a-f).

*n*, number of cases; SD, standard deviation; CI, confidence interval; T0, baseline; T1, post alignment; T2, post treatment, CPQ, Child perceptions questionnaire; OS, oral symptoms; FL, functional limitations; EW, emotional wellbeing; SW, social wellbeing; *U*, Mann–Whitney *U* test statistic; *r*, effect size for the Mann–Whitney *U* test.

^a^ *P* = .047.

^b^ *P* = .009.

^c^ *P* = .023.

^d^ *P* = .001.

^e^ *P* = .018.

^f^ *P* = .006.

Girls reported poorer OHRQoL and lower EW scores at T0, but showed greater EW improvements from T0 to T1 and T0 to T2 compared with boys (Table 2). Effect sizes for these differences were small to moderate. Statistically significant inter- and intragroup results were supported by the RSF16 sensitivity analyses (Table 2, Supplementary Table S2).

At T0, the ‘not applicable’ option was selected 79 times (1.71%), most frequently for item 6 (Bad breath, 13.6%), item 9 (Breathing through the mouth, 7.6%) and item 34 (Difficulty playing an instrument, 14.4%); all other items ranged between 0% and 3.0%.

At T1, the ‘not applicable’ option was selected 67 times (1.6%), most frequently for item 6 (bad breath, 8.40%), item 9 (breathing through the mouth, 6.72%), and item 34 (difficulty playing an instrument, 24.4%); all other items ranged between 0% and 2.52%.

At T2, the ‘not applicable’ option was selected 45 times (1.10%), most frequently for item 6 (bad breath, 10.0%), item 9 (breathing through the mouth, 3.33%), and item 34 (difficulty playing an instrument, 13.33%); all other items ranged between 0% and 1.67%.

### Feldmann’s questionnaires—aim 2

#### Prior to treatment

Results indicated high motivation and positive expectations towards treatment, with non-significant (NS) differences between treatment groups (Table 3). Across all collected questionnaires, 11 items (0.7%) were missed.

**Table 3:** Feldmann questionnaire results (mean, range, and percentiles) at T0, T1, and T2 by treatment group, with intergroup comparisons using Mann–Whitney *U* test.

							Percentiles								Percentiles											Percentiles		
		*n*	Mean	SD	Min	Max	25	50	75	*n*	Mean	SD	Min	Max	25	50	75	Mann–Whitney *U*			*n*	Mean	SD	Min	Max	25	50	75
Item		CB								PSLB								*U*	*r*	*P*	Total							
**T0**																												
Treatment motivation																												
1	Do your teeth bother you?	69	3.90	2.65	1	10	2	3	6	62	3.89	2.76	1	10	1	3	6	2115.00	0.01	.912	131	3.89	2.69	1	10	1	3	6
2	If it were possible, how much would you like to change the appearance of your teeth?	69	8.33	2.02	1	10	7	9	10	62	8.03	2.41	1	10	7	9	10	2028.50	0.05	.598	131	8.19	2.21	1	10	7	9	10
3	Do you think your teeth need straightening?	69	8.65	1.66	4	10	7	9	10	62	8.06	2.10	2	10	7	8.5	10	1812.50	0.14	.117	131	8.37	1.89	2	10	7	9	10
4	How motivated are you to have orthodontic treatment with braces?	69	8.61	2.05	1	10	8	10	10	62	8.00	2.21	2	10	7	9	10	1763.00	0.16	.067	131	8.32	2.14	1	10	7	9	10
5	Do you think orthodontic treatment will be good for your teeth?	69	8.97	1.14	6	10	8	9	10	62	8.74	1.44	4	10	8	9	10	2001.50	0.06	.506	131	8.86	1.29	4	10	8	9	10
6	Have you been properly informed about the orthodontic treatment?	69	8.58	2.35	1	10	8.5	9	10	62	8.76	1.96	1	10	8	10	10	2074.50	0.03	.748	131	8.66	2.17	1	10	8	10	10
7	Is it your own decision to undergo orthodontic treatment?	69	8.88	2.16	1	10	9	10	10	62	8.29	2.73	1	10	7	10	10	1929.50	0.10	.264	131	8.60	2.45	1	10	8	10	10
Treatment expectations																												
8	Do you think it is going to be difficult to wear braces?	69	4.49	1.91	1	8	3	5	6	62	4.84	2.23	1	10	3	5	6.25	1970.00	0.07	.431	131	4.66	2.06	1	10	3	5	6
9	Are you worried about having orthodontic treatment?	69	2.96	2.25	1	9	1	2	4	62	2.81	1.97	1	9	1	2	4	2103.00	0.01	.866	131	2.89	2.11	1	9	1	2	4
10	Are you worried about how you are going to look with braces on?	69	3.22	2.44	1	9	1	2	5	62	3.52	2.60	1	10	1	3	5.25	1978.00	0.07	.449	131	3.36	2.51	1	10	1	2	5
11	Have you ever been teased about the appearance of your teeth?	69	1.72	1.56	1	8	1	1	1.5	62	2.10	2.27	1	10	1	1	2	2025.00	0.06	.501	131	1.90	1.93	1	10	1	1	2
**T1**																												
1	Do you think orthodontic treatment is good for your teeth?	63	9.22	1.10	5	10	9	10	10	55	9.16	1.20	4	10	9	10	10	1686.00	0.03	.783	118	9.19	1.14	4	10	9	10	10
2	Do you think the braces are working as you expected?	63	9.22	1.74	1	10	9	10	10	55	9.09	1.09	6	10	8	9	10	1357.00	0.21	.**020**	118	9.16	1.47	1	10	9	10	10
3	Are you satisfied with the changes made during treatment so far?	63	9.17	1.36	4	10	9	10	10	55	9.38	0.87	7	10	9	10	10	1727.50	0.00	.976	118	9.27	1.16	4	10	9	10	10
4	Have you been properly informed during treatment?	63	8.78	1.61	3	10	8	9	10	55	8.91	1.55	2	10	8	9	10	1679.50	0.03	.762	118	8.84	1.58	2	10	8	9	10
5	Have you been properly cared for at your scheduled revisits?	63	9.46	0.93	6	10	9	10	10	55	9.64	0.65	8	10	9	10	10	1622.50	0.07	.462	118	9.54	0.81	6	10	9	10	10
6	Have you been properly cared for at your acute appointments?	63	9.51	1.18	5	10	10	10	10	55	9.47	1.14	3	10	9	10	10	1628.00	0.07	.471	118	9.49	1.15	3	10	9	10	10
7	Are you bothered by the braces?	63	4.49	2.53	1	10	3	4	7	55	4.47	2.50	1	10	3	4	6	1729.50	0.00	.988	118	4.48	2.51	1	10	3	4	7
8	Are the braces causing chafing?	63	3.71	2.47	1	10	2	3	6	55	3.64	2.25	1	10	2	3	5	1727.50	0.00	.979	118	3.68	2.36	1	10	2	3	5
9	Do you feel that the braces affect your mood?	63	2.05	1.80	1	9	1	1	2	55	2.07	1.54	1	7	1	1	3	1668.00	0.04	.702	118	2.06	1.68	1	9	1	1	3
10	Is it difficult to keep your teeth clean now that you have braces?	63	4.24	2.18	1	10	3	4	5	55	3.85	2.17	1	9	2	4	5	1536.00	0.10	.285	118	4.06	2.17	1	10	2.75	4	5
11	Do you feel that the braces have changed your appearance?	63	4.76	2.67	1	10	2	5	7	55	4.51	2.73	1	10	2	4	6	1626.50	0.05	.567	118	4.64	2.69	1	10	2	5	7
12	Have you been teased because you have braces?	63	1.24	0.76	1	6	1	1	1	55	1.25	0.95	1	7	1	1	1	1680.00	0.05	.712	118	1.25	0.85	1	7	1	1	1
13	Have you ever regretted starting orthodontic treatment	63	1.51	1.29	1	7	1	1	1	55	1.62	1.18	1	6	1	1	2	1626.50	0.07	.446	118	1.56	1.24	1	7	1	1	1.25
14	Are you tired of the treatment?	63	2.68	1.93	1	10	1	2	4	55	3.53	2.59	1	10	2	2	6	1457.50	0.14	.130	118	3.08	2.29	1	10	1	2	4
**T2**																												
Motivation, expectations, treatment outcome satisfaction																												
1	Do you think the orthodontic treatment has been good for your teeth?	62	9.77	0.58	7	10	10	10	10	56	9.57	0.83	6	10	9	10	10	1518.00	0.15	.105	118	9.68	0.71	6	10	10	10	10
2	Do you think the orthodontic treatment has worked as you expected?	62	9.61	0.93	5	10	10	10	10	56	9.45	0.95	6	10	9	10	10	1500.50	0.15	.108	118	9.53	0.94	5	10	9	10	10
3	Are you satisfied with the changes made during treatment?	62	9.82	0.43	8	10	10	10	10	56	9.45	0.89	6	10	9	10	10	1344.00	0.25	.**006**	118	9.64	0.71	6	10	9	10	10
4	Are you satisfied with the appearance of your teeth?	62	9.63	0.68	7	10	9	10	10	56	9.20	1.21	5	10	9	10	10	1390.00	0.20	.**029**	118	9.42	0.99	5	10	9	10	10
5	Have you ever regretted starting your orthodontic treatment?	62	1.60	1.40	1	9	1	1	2	56	1.66	1.40	1	8	1	1	2	1670.00	0.04	.656	118	1.63	1.39	1	9	1	1	2
6	If you had known what you know today before starting the treatment, would you have made the decision to undergo orthodontic treatment again?	62	6.55	4.06	1	10	1	9	10	56	6.79	3.97	1	10	1.25	9.50	10	1644.00	0.05	.597	118	6.66	4.00	1	10	1	9	10
Quality of care and attention																												
7	Have you been properly informed about the orthodontic treatment before starting your treatment?	62	8.66	1.73	3	10	8	9	10	56	8.91	1.52	4	10	8	10	10	1595.50	0.07	.418	118	8.78	1.63	3	10	8	9.88	10
8	Have you been properly informed during treatment?	62	9.32	0.99	6	10	9	10	10	56	9.29	1.09	6	10	9	10	10	1733.00	0.00	.987	118	9.31	1.03	6	10	9	10	10
9	Have you been properly cared for at your scheduled revisits	62	9.69	0.56	8	10	9	10	10	56	9.82	0.43	8	10	10	10	10	1561.50	0.12	.213	118	9.75	0.51	8	10	10	10	10
10	Have you been properly cared for at your acute appointments?	62	9.68	0.74	7	10	10	10	10	56	9.80	0.44	8	10	10	10	10	1685.00	0.04	.663	118	9.74	0.62	7	10	10	10	10
11	Did you receive information about how long the treatment would take?	62	8.21	2.07	2	10	7	9	10	56	8.02	2.11	1	10	7	9	9	1564.50	0.09	.345	118	8.12	2.08	1	10	7	9	10
Perceived pain and discomfort																												
12	Did you experience pain and discomfort when braces were bonded on your teeth?	62	5.89	2.59	1	10	3	6	8	56	5.13	2.49	1	10	3	5	7	1445.00	0.15	.114	118	5.53	2.56	1	10	3	6	7
13	Did you feel that the braces were causing chafing?	62	5.92	2.34	1	10	4	6	8	56	5.50	2.16	1	10	4	5,5	7	1549.50	0.09	.313	118	5.72	2.26	1	10	4	6	7
14	Did you experience pain and discomfort when the braces were removed?	62	3.02	2.16	1	9	1	2.25	4	56	3.04	2.13	1	8	1	2.75	4	1720.00	0.01	.931	118	3.03	2.14	1	9	1	2.50	4
15	Do you now experience pain and discomfort from your stabilization appliance?	62	2.15	1.91	1	8	1	1	2.25	56	2.25	2.08	1	9	1	1	2.75	1710.00	0.01	.877	118	2.19	1.99	1	9	1	1	2.25
16	Did you feel that the braces affected your mood?	62	2.95	2.41	1	9	1	2	4.25	56	3.05	2.54	1	10	1	2	4	1647.00	0.05	.621	118	3.00	2.46	1	10	1	2	4
Being teased and oral hygiene																												
17	Were you teased for wearing braces?	62	1.31	0.95	1	7	1	1	1	56	1.23	0.97	1	8	1	1	1	1690.50	0.04	.617	118	1.27	0.96	1	8	1	1	1
18	Did you find it difficult to keep your teeth clean while wearing braces?	62	4.26	2.34	1	10	3	4	5.25	56	4.04	2.37	1	10	2	4	5.75	1638.50	0.05	.597	118	4.15	2.35	1	10	2	4	5.25

End phrases at T0: ‘not at all’ and ‘completely’ for item 7, ‘not at all’ and ‘unbearable’ for items 8, 9, and 10, ‘not at all’ and ‘very much’ for all other items. End phrases at T1: ‘not at all’ and ‘completely’ for items 2 and 3, ‘never’ and ‘every day’ for item 13, ‘not at all’ and ‘very much’ for all other items. End phrases at T2: ‘not at all’ and ‘completely’ for items 2, 3, and 4, ‘never’ and ‘every day’ for item 5, ‘absolutely not’ and ‘very gladly’ for item 6, ‘not at all’ and ‘worst imaginable’ for items 12, 14, and 15, ‘not at all’ and ‘very much’ for all other items. Statistical significance was defined as *P* < 0.05. *P*-values in bold are statistically significant.

*n*, number of cases; SD, standard deviation; Min, minimum value; Max, maximum value; *P*, *P*-value; T0, baseline; T1, post alignment; T2, post treatment.

#### During treatment

Scores indicated that treatment was working as patients expected (item 2, Table 3) and that they did not regret starting treatment (item 13, Table 3). Teasing was minimal (item 12), and oral hygiene difficulties were rated low to mid-scale (item 10). The CB group scored higher on item 2 (‘Do you think the braces are working as you expected’) than the PSLB, although the effect size was small and clinical relevance may be limited (Table 3). Across the collected questionnaires, 12 items (0.7%) were missed.

#### Post treatment

Scores indicated positive experiences with treatment, quality of care and attention, and satisfaction with treatment outcomes (Table 3). Pain and discomfort were recalled at mid-scale, higher at bonding (item 12) than debonding (item 14), and low during stabilization appliance use (item 15). Teasing was minimal (item 17), and oral hygiene difficulties were scored low to mid-scale (item 18). The CB group reported slightly higher satisfaction with treatment changes and dental appearance (items 3 and 4), though the differences and the effect sizes were small (Table 3). Across collected questionnaires, 63 items (2.9%) were missed; of these, items 3, 4, and 6 each had only one missing response. Factors correlated to satisfaction with treatment outcome—aim 3.

#### Demographics, clinical characteristics and treatment effects

NS correlation was observed (Table 4).

**Table 4:** Correlation analysis between patient satisfaction with treatment outcome and demographics, clinical characteristics, and treatment outcomes; motivation and expectations reported at T0; motivation and expectations, quality of care and attention, teasing and oral hygiene reported at T1; and motivation and expectations, quality of care and attention, pain and discomfort, teasing and oral hygiene reported at T2, using Spearman rank correlation.

	Satisfaction with treatment outcome												
	If, before starting the treatment, you had known what you know today, would you have made the same decision to undergo orthodontic treatment?					Are you satisfied with the changes made during treatment?				Are you satisfied with the appearance of your teeth after treatment?			
Variable	*n*	*ρ*	*P*-value	95% CI		*ρ*	*P*-value	95% CI		*ρ*	*P*-value	95% CI	
**Correlation between demographics, clinical characteristics, treatment outcomes, and satisfaction with orthodontic treatment outcome.**													
Sex	118	0.106	.253	−0.081	0.286	0.001	.989	−0.185	0.187	−0.052	.579	−0.235	0.136
Age	118	−0.042	.654	−0.226	0.145	0.014	.882	−0.173	0.199	0.030	.747	−0.157	0.215
LII upper T0	114	−0.039	.682	−0.226	0.152	0.060	.525	−0.131	0.247	0.020	.830	−0.170	0.209
LII lower T0	116	−0.019	.836	−0.206	0.169	0.050	.592	−0.139	0.236	0.045	.632	−0.144	0.231
LII upper improvement	113	0.051	.591	−0.140	0.239	−0.050	.599	−0.238	0.141	−0.046	.630	−0.234	0.145
LII lower improvement	115	0.024	.798	−0.165	0.212	−0.051	.588	−0.237	0.139	−0.043	.649	−0.229	0.147
wPAR T0	118	−0.007	.936	−0.193	0.179	0.005	.955	−0.181	0.191	−0.018	.846	−0.203	0.169
wPAR T2	118	0.084	.366	−0.104	0.266	−0.046	.618	−0.230	0.141	−0.018	.845	−0.203	0.168
wPAR improvement %	118	−0.113	.222	−0.293	0.074	0.066	.481	−0.122	0.248	0.024	.797	−0.163	0.209
Total treatment time	116	0.043	.645	−0.146	0.229	−0.104	.266	−0.286	0.085	−0.027	.771	−0.214	0.161
Headache T0	117	−0.122	.190	−0.302	0.066	−0.133	.152	−0.312	0.055	−0.118	.206	−0.298	0.071
Headache T1	110	−0.020	.833	−0.212	0.173	−0.064	.509	−0.253	0.131	−0.205	.031	−0.383	−0.013
Headache T2	111	−0.013	.888	−0.205	0.179	−0.059	.540	−0.248	0.134	−0.046	.633	−0.236	0.147
IOTN DHC	118	−0.144	.119	−0.322	0.043	0.244	.008	0.061	0.4412	0.277	.002	0.096	0.440
Treatment group	118	0.049	.597	−0.138	0.233	−0.254	.005	−0.420	−0.071	−0.202	.028	−0.374	−0.017
**Correlations between treatment motivation, expectations reported at T0, and satisfaction with orthodontic treatment outcome**													
Treatment motivation													
Do your teeth bother you?	117	0.108	.246	−0.080	0.289	−0.031	.742	−0.216	0.157	0.002	.983	−0.185	0.189
If it were possible, how much would you like to change the appearance of your teeth?	117	0.110	.236	−0.078	0.291	0.112	.230	−0.077	0.293	−0.078	.406	−0.261	0.111
Do you think your teeth need straightening?	117	0.005	.958	−0.182	0.191	0.165	.076	−0.023	0.341	0.083	.372	−0.105	0.266
How motivated are you to have orthodontic treatment with braces?	117	0.147	.113	−0.041	0.325	0.130	.163	−0.058	0.309	0.049	.599	−0.139	0.234
Do you think orthodontic treatment will be good for your teeth?	117	−0.015	.872	−0.201	0.172	0.117	.210	−0.072	0.297	0.205	.027	0.019	0.377
Have you been properly informed about the orthodontic treatment?	117	0.016	.862	−0.171	0.202	0.131	.159	−0.057	0.310	0.131	.158	−0.057	0.311
Is it your own decision to undergo orthodontic treatment?	117	0.043	.646	−0.145	0.228	0.172	.063	−0.015	0.348	0.232	.012	0.048	0.402
Treatment expectations													
Do you think it is going to be difficult to wear braces?	117	0.101	.276	−0.087	0.283	−0.132	.156	−0.311	0.056	−0.298	.**001**	−0.459	−0.117
Are you worried about having orthodontic treatment?	117	−0.057	.538	−0.242	0.131	−0.021	.820	−0.207	0.166	−0.173	.062	−0.348	0.014
Are you worried about how you are going to look with braces on?	117	0.043	.645	−0.145	0.228	−0.007	.942	−0.193	0.180	−0.180	.052	−0.355	0.007
Have you ever been teased about the appearance of your teeth?	117	0.096	.302	−0.092	0.278	−0.011	.909	−0.197	0.176	−0.101	.277	−0.283	0.087
**Correlations between motivation and expectations, quality of care and attention, teasing and oral hygiene reported at T1, and satisfaction with orthodontic treatment outcome**													
Motivation and expectations, being teased, and quality of care and attention													
Do you think orthodontic treatment is good for your teeth?	111	0.033	.733	−0.160	0.223	0.160	.093	−0.033	0.342	0.042	.663	−0.151	0.232
Do you think the braces are working as you expected?	111	0.155	.103	−0.038	0.337	0.215	.023	0.025	0.391	0.068	.480	−0.126	0.256
Are you satisfied with the changes made during treatment so far?	111	0.129	.178	−0.064	0.313	0.253	.007	0.064	0.424	0.101	.294	−0.093	0.287
Have you been properly informed during treatment?	111	0.031	.744	−0.161	0.222	0.181	.057	−0.011	0.360	0.078	.418	−0.116	0.266
Have you been properly cared for at your scheduled revisits?	111	0.114	.234	−0.080	0.299	0.154	.107	−0.039	0.336	0.060	.529	−0.133	0.249
Have you been properly cared for at your acute appointments?	111	0.068	.477	−0.125	0.257	0.067	.486	−0.127	0.255	−0.017	.858	−0.208	0.175
Are you bothered by the braces?	111	−0.139	.147	−0.322	0.055	−0.090	.346	−0.277	0.103	−0.157	.100	−0.339	0.036
Are the braces causing chafing?	111	0.130	.173	−0.063	0.314	−0.076	.425	−0.264	0.117	−0.127	.184	−0.311	0.066
Do you feel that the braces affect your mood?	111	−0.161	.090	−0.343	0.031	−0.141	.141	−0.324	0.052	−0.248	.009	−0.420	−0.059
Is it difficult to keep your teeth clean now that you have braces?	111	−0.091	.345	−0.277	0.103	−0.049	.612	−0.238	0.144	−0.238	.012	−0.411	−0.048
Do you feel that the braces have changed your appearance?	111	−0.038	.694	−0.228	0.155	−0.106	.267	−0.292	0.087	−0.207	.029	−0.384	−0.016
Have you been teased because you have braces?	111	−0.090	.350	−0.277	0.104	0.024	.802	−0.169	0.215	−0.053	.580	−0.242	0.140
Have you ever regretted starting orthodontic treatment?	111	−0.174	.067	−0.354	0.018	−0.100	.294	−0.287	0.093	−0.038	.695	−0.228	0.155
Are you tired of the treatment?	111	0.332	**<**.**001**	0.149	0.492	−0.093	.331	−0.280	0.100	−0.151	.113	−0.333	0.042
**Correlation between reported motivation and expectations, quality of care and attention, pain and discomfort, teasing and oral hygiene reported at T2, and satisfaction with orthodontic treatment outcome**													
Motivation and expectations													
Do you think the orthodontic treatment has been good for your teeth?	118	0.034	.714	−0.153	0.219	0.525	**<**.**001**	0.376	0.648	0.342	**<**.**001**	0.167	0.496
Do you think the orthodontic treatment has worked as you expected?	118	0.061	.513	−0.127	0.244	0.330	**<**.**001**	0.153	0.486	0.273	.003	0.092	0.437
Have you ever regretted starting your orthodontic treatment?	118	−0.124	.181	−0.303	0.063	−0.256	.005	−0.422	−0.073	−0.245	.007	−0.413	−0.062
Quality of care and attention													
Have you been properly informed about the orthodontic treatment before starting your treatment?	118	0.065	.482	−0.122	0.248	0.095	.304	−0.092	0.277	0.157	.090	−0.030	0.333
Have you been properly informed during treatment?	118	−0.007	.938	−0.193	0.179	0.278	.002	0.097	0.441	0.071	.445	−0.117	0.254
Have you been properly cared for at your scheduled revisits?	118	0.008	.933	−0.178	0.194	0.199	.031	0.014	0.371	0.210	.023	0.025	0.381
Have you been properly cared for at your acute appointments?	118	0.069	.456	−0.118	0.252	0.239	.009	0.055	0.407	0.228	.013	0.044	0.397
Did you receive information about how long the treatment would take?	118	−0.119	.201	−0.298	0.069	0.168	.069	−0.019	0.343	0.073	.430	−0.114	0.256
Perceived pain and discomfort													
Did you experience pain and discomfort when braces were bonded on your teeth?	118	−0.030	.746	−0.215	0.157	−0.031	.743	−0.215	0.156	−0.157	.090	−0.333	0.030
Did you feel that the braces were causing chafing?	118	−0.022	.809	−0.208	0.164	−0.175	.057	−0.350	0.011	−0.221	.016	−0.390	−0.036
Did you experience pain and discomfort when the braces were removed?	118	0.009	.919	−0.177	0.195	−0.005	.959	−0.191	0.181	−0.155	.095	−0.331	0.032
Do you now experience pain and discomfort from your stabilization appliance?	118	0.020	.834	−0.167	0.205	−0.086	.354	−0.268	0.101	−0.158	.088	−0.334	0.029
Did you feel that the braces affected your mood?	118	0.019	.837	−0.167	0.204	−0.156	.092	−0.332	0.031	−0.149	.108	−0.326	0.038
Teasing and oral hygiene													
Were you teased for wearing braces?	118	−0.077	.406	−0.259	0.110	−0.124	.179	−0.303	0.063	−0.174	.059	−0.349	0.012
Did you find it difficult to keep your teeth clean while wearing braces?	118	0.008	.929	−0.178	0.194	−0.084	.368	−0.265	0.104	−0.277	.002	−0.440	−0.095

Statistical significance with Bonferroni-adjusted thresholds was set to *P* < .0011 for analyses with 45 comparisons (demographics, clinical characteristics and treatment outcomes; and questionnaire results at T2), *P* < .0015 for 33 comparisons (questionnaire results at T0), and *P* < .0012 for 42 comparisons (questionnaire results at T1). *P*-values in bold are statistically significant.

*n*, number of cases; *ρ* = Spearman’s rho; CI, confidence interval; LII, Little’s Irregularity Index; T0, baseline; T1, post alignment; T2, post treatment; wPAR, weighted Peer Assessment Rating; IOTN, Index of Orthodontic Treatment Need.

#### Factors assessed prior to treatment

A weak negative correlation was observed for ‘Do you think it is going to be difficult to wear braces’ (Table 4).

#### Factors assessed during treatment

A moderate positive correlation was observed for ‘Are you tired of the treatment’ (Table 4).

#### Factors assessed post treatment

A strong positive correlation was observed for ‘Do you think the orthodontic treatment has been good for your teeth’, and a moderate positive correlation for ‘Do you think the orthodontic treatment has worked as you expected’ (Table 4).

Sensitivity analyses suggested that the omission of the two CPQ11-14 items had minimal impact on the effect sizes and statistical significance (Supplementary Table S2). Similarly, analyses using complete questionnaires showed that imputations had minimal effect on the effect sizes and the statistical significance of the CPQ11-14 and Feldmann’s questionnaire (Supplementary Table S3).

### Harms

Minor harms commonly associated with orthodontic treatment were observed in both groups, such as plaque accumulation, gingivitis, chafing, and minor root shortening.

## Discussion

This is the first comprehensive RCT on OHRQoL during and after orthodontic treatment with PSLB or CB in patients with crowding, offering valuable insights into patient experience. The findings demonstrated a decline in OHRQoL during active treatment, possibly reflecting burdens of fixed appliance treatment, followed by a post-treatment improvement beyond baseline. The differences between systems were small, suggesting comparable effectiveness in terms of the assessed patient-reported outcomes. Girls reported poorer baseline OHRQoL but greater improvement, resulting in NS differences at T2. Patients were generally motivated and not worried about starting orthodontic treatment. Although OHRQoL declined at T1, motivation for treatment remained high, and patients’ expectations were met. At T2, patients reported high satisfaction and, importantly, did not regret starting treatment. Our findings suggest that patients similar to the sample studied should be prepared for possible temporary OHRQoL reductions, while being informed of the potential long-term benefits.

Inclusion of QoL measures in research is recommended, as patient perception is essential for a comprehensive understanding of treatment effects [41]. Baseline CPQ11-14 scores indicated better OHRQoL than those reported by Jokovic *et al*. [13], but were similar to findings from European studies involving children and adolescents with mixed malocclusions [14, 24]. Due to the 35-item format, direct score comparisons should be interpreted with caution. Nonetheless, even when applying the RSF16 analysis, baseline values remained lower than those reported by Jokovic *et al*. [39].

Post-treatment OHRQoL improved beyond baseline, consistent with previous research [14, 15]. Some studies report improvements reaching or exceeding baseline scores as early as 6 months into treatment [23, 42]. Such variations may reflect inclusion of patients with different malocclusion types, treatment protocols, or sociocultural influences. A qualitative study from Brazil linked braces to social status and identity among adolescents [43]. Wearing the fixed appliance could in itself be perceived as aesthetically positive, and economic aspects of treatment were suggested to be related to a culturally established link between orthodontic appliances and social status [43]. These factors may partly explain the rapid OHRQoL improvements reported in some studies. In contrast, Swedish orthodontic treatment is publicly funded for children and adolescents with an objective treatment need (equivalent to IOTN DHC 4 and 5), reducing socioeconomic impact of wearing braces. Although disparities have been reported [44], all patients in the present study received publicly funded treatment. Within this context, improvements in aesthetics, function, self-esteem, and confidence likely contributed to the observed OHRQoL gains [45].

Both significant and NS associations between malocclusion and OHRQoL have been reported [46–48]. In the present study, strict eligibility criteria ensured inclusion of adolescents with crowding and dental irregularity, producing a cohort with pronounced anterior contact point displacements (LII, Table 1) and baseline CPQ11-14 scores comparable to those of Austrian adolescents with severe contact point displacement (IOTN 4d) [24]. The results support previous research linking aesthetic-zone malocclusions to reduced OHRQoL, particularly in the EW and SW domains [3,4, 49] . However, children with similar dental aesthetic impairments may perceive treatment need differently, and sociocultural variations in how dental aesthetics and malocclusions are interpreted may limit generalizability of results [26].

Both treatment groups reported a statistically significant improvement in OHRQoL at T2, with NS intergroup differences in total CPQ11-14 scores, consistent with previous cohort studies [17, 19] and RCTs [18, 20]. These studies vary in population characteristics, age, assessment timing, and use different dPROMs compared with the present study, supporting the observed lack of intergroup differences.

The largest CPQ score changes were observed in the FL and OS domains, aligning with previous research [24]. Deterioration at T1 was followed by improvement beyond baseline at T2, likely reflecting burdens of wearing braces, such as pain and discomfort, food entrapment and prolonged eating time. Although orthodontic pain can affect OHRQoL [50], and lower pain scores with less analgesic use have been reported in the PSLB group [16], the OS domain improved more in the CB group during treatment. However, the effect size was small, and the difference in mean score was 1.28 (Table 2). Since we have no minimal detectable change for this sample, the clinical relevance of the difference is uncertain.

To minimize the risk of conflating treatment effects with immediate procedural discomfort, the questionnaires were distributed prior to scheduled treatments [41]. Therefore, the worsened OHRQoL at T1, does not reflect the high pain levels and intergroup differences reported post bonding, as pain levels were low before insertion of the 0.019 × 0.025 archwire [16]. This suggests that factors other than pain contributed to the increased CPQ11-14 scores at T1. Importantly, the T1 scores represent a single time point, so CPQ score variations during other periods, such as those characterized by intense pain, remain unknown. More frequent assessments and qualitative methods could provide deeper insights, though it would increase patient burden.

The EW and SW domains improved throughout treatment, consistent with previous research [24], and have recently been linked to growing social acceptance and popularity of braces, promoting normalization and faster psychological adaptation [50]. Impairments in both EW and SW have been associated with dental crowding in children and adolescents [4]. The impact of malocclusion on OHRQoL varies across age groups, and typically becomes noticeable around eight years of age, with younger children and adolescents reporting less impact than older peers [25]. Aesthetically impairing malocclusions have further been associated with feelings of loneliness[51], and girls generally report a stronger association between subjective treatment need and OHRQoL [52]. In the present study, girls reported poorer baseline OHRQoL, contrasting recent German findings [4, 24]. The differences were observed in the EW domain and had small to moderate effect sizes. Post treatment, the differences were NS.

At T0, patients reported high motivation and positive expectations towards orthodontic treatment. Aesthetic-related items in the motivation domain received high scores, and overall pattern of scores was consistent with previous research [28, 53]. Aesthetic concerns are a primary motivation for seeking treatment [15, 54]. Interestingly, concern about appearance while wearing a fixed appliance was relatively low at T0, aligning with recent UK findings and possibly reflecting changing attitudes over time [29, 50]. Earlier Swedish research involving adolescents reported appearance related concerns as an argument against fixed appliance treatment [55].

During treatment (T1), patients reported high scores for quality of care and attention, and low scores for regret about starting treatment and treatment fatigue, indicating sustained motivation and a positive experience. The CB group scored slightly higher on the item ‘Do you think the braces are working as you expected’, although the effect size was small, so the clinical relevance is unclear. Both groups generally perceived treatment to have met their expectations.

At T2, satisfaction with treatment outcomes was generally high, and patients did not regret having started treatment. Notably, the wording of the satisfaction related item ‘If, before starting the treatment, you had known what you know today, would you have made the same decision to undergo orthodontic treatment’, may be difficult to interpret and risks conflating the burden of study participation with the treatment experience. Since overall satisfaction scores were high while this item was scored comparatively lower, the results should be interpreted with this consideration in mind.

In the exploratory analyses, a negative correlation was observed between satisfaction and baseline expectations of treatment difficulty, as well as greater satisfaction when the treatment has worked as expected. Greater patient satisfaction has been suggested when expectations align with outcomes, although with weak evidence [56], and a strong influence from doctor–patient relationship has been reported [57]. This emphasizes the importance of patient–caregiver communication to ensure realistic patient expectations, as these may not necessarily align with reality [58]. Meanwhile, a previously reported link between motivation and satisfaction with treatment outcome was not observed in the present study [53]. The absence of significant correlations may reflect an insufficient sample size. Therefore, results should be interpreted with knowledge of type II error in mind.

A moderate positive correlation was found between treatment fatigue and satisfaction. This association could be discussed in relation to effort justification and cognitive dissonance theories, which propose that individuals who perceive treatment as more demanding may increase the value attributed to the outcome [59].

The CB group reported somewhat higher satisfaction with dental appearance and with the changes made during treatment, although the difference was small (0.4) with a small effect size. Intergroup differences could be explored further in future qualitative research, allowing patient reflections and a more comprehensive understanding of the findings. Additionally, the exploratory correlation results may serve as a basis for developing future hypotheses.

### Strengths and limitations

A strength is the use of validated, reliable, and age appropriate instruments suitable for longitudinal research [13, 30, 60]. The CPQ11-14 was the most frequently used instrument at CROWDIT initiation, and has been validated for adolescents transitioning into adulthood [60]. The timing of questionnaire distribution reduced the risk of conflating the dPROs with immediate effects of interventions [41].

The volume of items may have contributed to missing responses. A digital format might have facilitated completion. Although a short form questionnaire may reduce patient burden, full versions cover more information [39]. Patients were given time at the clinic and encouraged to reflect on their responses, which likely improved response quality. Frequent ‘not applicable’ responses to certain items may indicate their limited relevance for this sample.

Two CPQ11-14 items were omitted, affecting SW and total CPQ score, which prompted sensitivity analyses. Analyses using the CPQ11-14 RSF16 (Supplementary Table S2) and complete questionnaires (Supplementary Table S3) showed minimal impact of item omission and imputation of missing responses on effect sizes or statistical significance. Because translated and culturally adapted CPQ11-14 versions may differ somewhat, e.g. in the number of items [13, 36, 61], comparing changes over time may be more informative than comparing absolute scores.

No *a priori* sample size calculation was feasible, which is a limitation of the study. The observed effect sizes indicate that a larger sample size would be needed to detect intergroup differences. Non-significant results should therefore not be interpreted as confirmation of the null hypothesis.

An untreated control group was ethically infeasible given CROWDIT’s longitudinal design. Awareness of study participation could have influenced patient behaviour (Hawthorne effect), introducing potential bias [62]. Caries status can also influence OHRQoL. However, because good oral health and hygiene are prerequisites for starting orthodontic treatment, caries likely had little impact in this study.

### Generalizability

The multicentre design enhances generalizability, mainly to Swedish and possibly northern European adolescents with crowding treated with fixed appliances. Sociocultural factors may influence perceptions over time, highlighting the relevance of studies like this.

### Future studies

Qualitative interviews could deepen the understanding of adolescents’ experiences, satisfaction, and OHRQoL.

## Conclusion

This RCT evaluated and compared OHRQoL, motivation, expectations, and satisfaction with treatment outcomes in adolescents with dental crowding treated without extractions, using either a conventional or a PSLB system.

The results indicate benefits of fixed appliance treatment for adolescents similar to those in the studied sample under the described circumstances. Patients reported improved OHRQoL and high satisfaction with treatment outcomes, including enhanced dental appearance, and no regret about having started treatment. The results also highlight the importance of patient-caregiver communication, the value of incorporating dPROs in orthodontic practice, and may be used to guide future study design and sample size calculations.

## Supplementary Material

cjag040_Supplementary_Data

## Data Availability

The data underlying this article will be shared on reasonable request to the corresponding author.

## References

[1] Lombardo G, Vena F, Negri P et al Worldwide prevalence of malocclusion in the different stages of dentition: a systematic review and meta-analysis. Eur J Paediatr Dent 2020;21:115–22. 10.23804/ejpd.2020.21.02.05 32567942

[2] Turner S, Harrison JE, Sharif FNJ et al Orthodontic treatment for crowded teeth in children. Cochrane Database Syst Rev 2021;12:CD003453. 10.1002/14651858.CD003453.pub2 34970995 PMC8786262

[3] Dimberg L, Arnrup K, Bondemark L. The impact of malocclusion on the quality of life among children and adolescents: a systematic review of quantitative studies. Eur J Orthod 2015;37:238–47. 10.1093/ejo/cju046 25214504

[4] Von Laffert A, Riemekasten S, Kieß W et al Influence of upper anterior teeth crowding on the quality of life of children and adolescents. J Orofac Orthop 2025; 10.1007/s00056-025-00595-w PMC13503400 40828393

[5] Cinelli F, Paolini E, Nieri M et al Bullying, cyberbullying, self-esteem, psychological distress and relationship with oral health related quality of life: a cross-sectional survey in adolescents. Eur J Orthod 2025;47:cjaf058. 10.1093/ejo/cjaf058 40810338 PMC12351380

[6] John MT . Health outcomes reported by dental patients. J Evid Based Dent Pract 2018;18:332–5. 10.1016/j.jebdp.2018.09.001 30514446

[7] Tao Z, Zhao T, Ngan P et al The use of dental patient-reported outcomes among randomized controlled trials in orthodontics: a methodological study. J Evid Based Dent Pract 2023;23:101795. 10.1016/j.jebdp.2022.101795 36707165

[8] Huang X, Tao Z, Ngan P et al The use of dental patient-reported outcomes among comparative observational studies in orthodontics: a methodological study. J Evid Based Dent Pract 2024;24:101956. 10.1016/j.jebdp.2023.101956 38401953

[9] Gift HC, Atchison KA. Oral health, health, and health-related quality of life. Med Care 1995;33:Ns57–77. 10.1097/00005650-199511001-00008 7475433

[10] Al Shamrany M . Oral health-related quality of life: a broader perspective. East Mediterr Health J 2006;12:894–901. 17333837

[11] Locker D, Allen F. What do measures of ‘oral health-related quality of life’ measure? Community Dent Oral Epidemiol 2007;35:401–11. 10.1111/j.1600-0528.2007.00418.x 18039281

[12] Klages U, Claus N, Wehrbein H et al Development of a questionnaire for assessment of the psychosocial impact of dental aesthetics in young adults. Eur J Orthod 2006;28:103–11. 10.1093/ejo/cji083 16257989

[13] Jokovic A, Locker D, Stephens M et al Validity and reliability of a questionnaire for measuring child oral-health-related quality of life. J Dent Res 2002;81:459–63. 10.1177/154405910208100705 12161456

[14] Jaeken K, Cadenas de Llano-Pérula M, Lemiere J et al Reported changes in oral health-related quality of life in children and adolescents before, during, and after orthodontic treatment: a longitudinal study. Eur J Orthod 2019;41:125–32. 10.1093/ejo/cjy035 29917078

[15] Yassir YA, McIntyre GT, Bearn DR. The impact of labial fixed appliance orthodontic treatment on patient expectation, experience, and satisfaction: an overview of systematic reviews. Eur J Orthod 2020;42:223–30. 10.1093/ejo/cjz043 31147683

[16] Johansson K, Matilainen LB, Wiaderny M et al Self-reported pain during different phases of orthodontic treatment with fixed appliance: a multi-centre randomized controlled trial in adolescents with crowding. Orthod Craniofac Res 2024;27:560–71. 10.1111/ocr.12771 38389292

[17] Barrera-Chaparro JP, Plaza-Ruíz SP, Parra KL et al Orthodontic treatment need, the types of brackets and the oral health-related quality of life. Dent Med Probl 2023;60:287–94. 10.17219/dmp/151577 37458398

[18] Othman SA, Mansor N, Saub R. Randomized controlled clinical trial of oral health-related quality of life in patients wearing conventional and self-ligating brackets. Korean J Orthod 2014;44:168–76. 10.4041/kjod.2014.44.4.168 25133131 PMC4130912

[19] Zhou Y, Zheng M, Lin J et al Self-ligating brackets and their impact on oral health-related quality of life in Chinese adolescence patients: a longitudinal prospective study. ScientificWorldJournal 2014;2014:352031. 10.1155/2014/352031 25202720 PMC4151365

[20] Lai TT, Chiou JY, Lai TC et al Oral health-related quality of life in orthodontic patients during initial therapy with conventional brackets or self-ligating brackets. J Dent Sci 2017;12:161–72. 10.1016/j.jds.2016.12.003 30895043 PMC6395242

[21] Petrén S, Bondemark L, Sonesson M et al A systematic review considering the risk of bias in orthodontic RCTs over 55 years. Eur J Orthod 2025;47:cjaf083. 10.1093/ejo/cjaf083 41121579 PMC12540042

[22] Vidigal MTC, Mesquita CM, de Oliveira MN et al Impacts of using orthodontic appliances on the quality of life of children and adolescents: systematic review and meta-analysis. Eur J Orthod 2022;44:359–68. 10.1093/ejo/cjac003 35201317

[23] Fahd CG, Castro GG, Costa ACS et al Oral health-related quality of life among adolescents in the first 6 months of fixed orthodontic therapy. Int J Environ Res Public Health 2023;20:7110. 10.3390/ijerph20237110 38063540 PMC10706226

[24] Schwarz L, Ossmann V, Ritschl V et al Influence of malocclusion on OHRQoL in adolescents in initial orthodontic treatment phase. Clin Oral Investig 2024;28:286. 10.1007/s00784-024-05689-0 PMC11058762 38684531

[25] Kragt L, Dhamo B, Wolvius EB et al The impact of malocclusions on oral health-related quality of life in children-a systematic review and meta-analysis. Clin Oral Investig 2016;20:1881–94. 10.1007/s00784-015-1681-3 PMC5069349 26635095

[26] Alrashed M, Alqerban A. The relationship between malocclusion and oral health-related quality of life among adolescents: a systematic literature review and meta-analysis. Eur J Orthod 2021;43:173–83. 10.1093/ejo/cjaa051 33009547

[27] Chong H, Peh J, Weir T et al Patient experiences with clear aligners: a scoping review. Eur J Orthod 2025;47:cjaf017. 10.1093/ejo/cjaf017 40237388 PMC12001029

[28] Feldmann I . Satisfaction with orthodontic treatment outcome. Angle Orthod 2014;84:581–7. 10.2319/093013-710.1 24423202 PMC8650456

[29] Longstaff S, Davies K, Benson P. Exploring 10–15-year-old patients’ perspectives of fixed orthodontic treatment. J Orthod 2021;48:110–7. 10.1177/1465312520981077 33573439

[30] Feldmann I, List T, John MT et al Reliability of a questionnaire assessing experiences of adolescents in orthodontic treatment. Angle Orthod 2007;77:311–7. 10.2319/0003-3219(2007)077[0311:Roaqae]2.0.Co;2 17319767

[31] Liu C, Seehra J, Cobourne MT. Longitudinal bibliometric investigation of published orthodontic research 2013–23: are we investigating patient-reported outcomes? Eur J Orthod 2024;46:cjae042. 10.1093/ejo/cjae042 39162175

[32] Hopewell S, Chan AW, Collins GS et al CONSORT 2025 statement: updated guideline for reporting randomised trials. Lancet 2025;405:1633–1640. 10.1016/s0140-6736(25)00672-5 40245901

[33] Shaw WC, Richmond S, O'Brien KD et al The use of occlusal indices: a European perspective. Am J Orthod Dentofacial Orthop 1995;107:1–10. 10.1016/s0889-5406(95)70151-6 7817954

[34] Malmö University . *Oral health country/area profile project/methods and indices Silness-Löe index*. https://capp.mau.se/Methods-and-Indices/?msclkid=6636f1e8b0ce11ec828439401687d01b (19 October, 2022, date last accessed).

[35] Matilainen LB, Johansson K, Wiaderny M et al Treatment effects and treatment time in adolescents with crowded and displaced teeth treated with fixed appliance systems without extractions: a multi-centre randomised controlled trial. Orthod Craniofac Res 2025;28:929–42. 10.1111/ocr.70005 40698883 PMC12603669

[36] Kallunki J, Bondemark L, Paulsson L. Early headgear activator treatment of class II malocclusion with excessive overjet: a randomized controlled trial. Eur J Orthod 2021;43:639–47. 10.1093/ejo/cjaa073 33274388

[37] Little RM . The irregularity index: a quantitative score of mandibular anterior alignment. Am J Orthod 1975;68:554–63. 10.1016/0002-9416(75)90086-x 1059332

[38] Richmond S, Shaw WC, O'Brien KD et al The development of the PAR Index (Peer Assessment Rating): reliability and validity. Eur J Orthod 1992;14:125–39. 10.1093/ejo/14.2.125 1582457

[39] Jokovic A, Locker D, Guyatt G. Short forms of the Child Perceptions Questionnaire for 11–14-year-old children (CPQ11-14): development and initial evaluation. Health Qual Life Outcomes 2006;4:4. 10.1186/1477-7525-4-4 16423298 PMC1368964

[40] Hopewell S, Chan AW, Collins GS et al CONSORT 2025 Statement: updated guideline for reporting randomised trials. BMJ 2025;388:e081123. 10.1136/bmj-2024-081123 PMC11995449 40228833

[41] Cunningham SJ, Hunt NP. Quality of life and its importance in orthodontics. J Orthod 2001;28:152–8. 10.1093/ortho/28.2.152 11395531

[42] Zhang M, McGrath C, Hägg U. Changes in oral health-related quality of life during fixed orthodontic appliance therapy. Am J Orthod Dentofacial Orthop 2008;133:25–9. 10.1016/j.ajodo.2007.01.024 18174067

[43] Barbosa de Almeida A, Leite ICG, Alves da Silva G. Brazilian adolescents’ perception of the orthodontic appliance: a qualitative study. Am J Orthod Dentofacial Orthop 2019;155:490–7. 10.1016/j.ajodo.2018.05.020 30935604

[44] Göranson E, Sonesson M, Dimberg L et al Equality of specialist orthodontic care for adolescents in the Swedish public dental service: a cohort study. BMC Oral Health 2025;25:841. 10.1186/s12903-025-06220-x 40437452 PMC12121215

[45] AlQuraini N, Shah R, Cunningham SJ. Perceptions of outcomes of orthodontic treatment in adolescent patients: a qualitative study. Eur J Orthod 2019;41:294–300. 10.1093/ejo/cjy071 30689803

[46] Kunz F, Platte P, Keß S et al Correlation between oral health-related quality of life and orthodontic treatment need in children and adolescents-a prospective interdisciplinary multicentre cohort study. J Orofac Orthop 2018;79:297–308. 10.1007/s00056-018-0142-4 29947814

[47] Vedovello SAS, de Carvalho ALM, de Azevedo LC et al Impact of anterior occlusal conditions in the mixed dentition on oral health-related quality-of-life item levels. Angle Orthod 2020;90:564–70. 10.2319/090219-571.1 33378500 PMC8028459

[48] Theodoridou MZ, Herclides A, Lamnisos D. Need for orthodontic treatment and oral health-related quality of life in children and adolescents—a systematic review. Community Dent Health 2024;41:5–13. 10.1922/CDH_00125Theodoridou09 37988672

[49] Kallunki J, Sollenius O, Paulsson L et al Oral health-related quality of life among children with excessive overjet or unilateral posterior crossbite with functional shift compared to children with no or mild orthodontic treatment need. Eur J Orthod 2019;41:111–6. 10.1093/ejo/cjy033 29878165

[50] Schmahl T, Steinhäuser J, Wewetzer L et al Impact of fixed orthodontic appliance treatment on children’s mental health, quality of life and social context: a scoping review. Patient Prefer Adherence 2025;19:1273–82. 10.2147/ppa.S509901 40330537 PMC12054534

[51] DiBiase A, Cox Z, Rea M et al Malocclusion and peer relationships in school children aged 10–14 years in the United Kingdom: a cross-sectional study. Am J Orthod Dentofacial Orthop 2025;168:435–50. 10.1016/j.ajodo.2025.04.017 40407766

[52] Kragt L, Jaddoe V, Wolvius E et al The association of subjective orthodontic treatment need with oral health-related quality of life. Community Dent Oral Epidemiol 2017;45:365–71. 10.1111/cdoe.12299 28370341 PMC5501354

[53] Li W, Wang S, Zhang Y. Relationships among satisfaction, treatment motivation, and expectations in orthodontic patients: a prospective cohort study. Patient Prefer Adherence 2016;10:443–7. 10.2147/ppa.S101825 27110100 PMC4831590

[54] Prado LH, Previato K, Delgado RZR et al Adolescents’ perception of malocclusion, their motivations, and expectations concerning the orthodontic treatment. Is it all about attractiveness? A qualitative study. Am J Orthod Dentofacial Orthop 2022;161:e345–52. 10.1016/j.ajodo.2021.10.014 35031194

[55] Trulsson U, Strandmark M, Mohlin B et al A qualitative study of teenagers’ decisions to undergo orthodontic treatment with fixed appliance. J Orthod 2002;29:197–204; discussion 195. 10.1093/ortho/29.3.197 12218197

[56] Yao J, Li DD, Yang YQ et al What are patients’ expectations of orthodontic treatment: a systematic review. BMC Oral Health 2016;16:19. 10.1186/s12903-016-0182-3 26884053 PMC4756524

[57] Keles F, Bos A. Satisfaction with orthodontic treatment. Angle Orthod 2013;83:507–11. 10.2319/092112-754.1 23181757 PMC8763066

[58] Aulu AM, Pikkusaari T, Rice D et al Expectations of orthodontic treatment among 7–12 year-old children—a cross sectional study. Acta Odontol Scand 2025;84:377–85. 10.2340/aos.v84.43910 40528488 PMC13063825

[59] Inzlicht M, Shenhav A, Olivola CY. The effort paradox: effort is both costly and valued. Trends Cogn Sci 2018;22:337–49. 10.1016/j.tics.2018.01.007 29477776 PMC6172040

[60] Chapman RA, Thomson WM, Broadbent JM. Using the Child Perceptions Questionnaire with young adults. Community Dent Oral Epidemiol 2023;51:1225–31. 10.1111/cdoe.12887 37291732

[61] Bekes K, John MT, Zyriax R et al The German version of the Child Perceptions Questionnaire (CPQ-G11-14): translation process, reliability, and validity in the general population. Clin Oral Investig 2012;16:165–71. 10.1007/s00784-010-0496-5 21210166

[62] Abdulraheem S, Bondemark L. Hawthorne effect reporting in orthodontic randomized controlled trials: truth or myth? Blessing or curse? Eur J Orthod 2018;40:475–9. 10.1093/ejo/cjx089 29186392

