# Supplementary material for: Oral health-related quality of life, experience and satisfaction in adolescents treated for dental crowding with self-ligating or conventional fixed appliances: a multicentre randomized controlled trial
Source: Eur J Orthod. 2026 Jun 8;48(4):cjag040. doi: 10.1093/ejo/cjag040 (PMC13244279; doi:10.1093/ejo/cjag040)
Supplement: cjag040_Supplementary_Data [file cjag040_supplementary_data.zip › S1. Missing questionnaires.docx]

| **Supplementary Table 1**. Reasons for missing questionnaires. | | | | | |
| --- | --- | --- | --- | --- | --- |
| Number collected at time point | Missing (CB/PSLB) | Unintentionally not administered | Patient discontinued treatment (n) | Patient moved |  |
| CPQ11-14 | | | | | |
| 119 at T1 | 13 (6/7) | 6 | 5^a^ | 2 |  |
| 120 at T2 | 12 (5/7) | 5 | 5^a^ | 2 |  |
| Feldmann’s questionnaires | | | | | |
| 131 at T0 | 1 (1/0) | 1 |  |  |  |
| 118 at T1 | 14 (7/7) | 7 | 5^a^ | 2 |  |
| 119 at T2 | 13 (7/6) | 7 | 5^a^ | 2 |  |
| ^a^ trauma (1), lack of oral hygiene (1), patient discontinued treatment/did not cooperate (2), needed extractions (1) | | | | | |
