# Supplementary material for: Oral health-related quality of life, experience and satisfaction in adolescents treated for dental crowding with self-ligating or conventional fixed appliances: a multicentre randomized controlled trial
Source: Eur J Orthod. 2026 Jun 8;48(4):cjag040. doi: 10.1093/ejo/cjag040 (PMC13244279; doi:10.1093/ejo/cjag040)
Supplement: cjag040_Supplementary_Data [file cjag040_supplementary_data.zip › S2. Results CPQ 11-14 change and RSF16 sensitivity analysis.docx]

| **Supplementary table 2**. CPQ11-14 scores for the total sample, each treatment group, and boys and girls. Score changes from T0 to T1 and T0 to T2 using the Wilcoxon signed-ranks test. | | | | | | | | | | | | | | |
| --- | --- | --- | --- | --- | --- | --- | --- | --- | --- | --- | --- | --- | --- | --- |
| **Total Sample** | | | | | | | | | | | | | | |
| **Variable** | **n** | | **Mean** | **SD** |  | | **Median** | **Percentiles** | |  |  | **Sensitivity analysis**  **RSF16** | | |
|  |  |  |  |  | **Min** | **Max** |  | **25** | **75** | **r** | **p** | **Median** | **r** | **p** |
| CPQT0 | 132 | 17.79 | | 12.12 | 3.00 | 61.00 | 15.00 | 8.25 | 23.75 |  |  | 9.00 |  |  |
| CPQT1 | 119 | 20.18 | | 11.77 | 3.00 | 59.00 | 17.00 | 11.00 | 27.00 |  |  | 10.00 |  |  |
| CPQT2 | 120 | 11.53 | | 8.39 | 0.00 | 55.00 | 9.00 | 6.00 | 14.75 |  |  | 5.00 |  |  |
| CPQ T0 to T1 | 119 | -2.56 | | 12.92 | -34.00 | 39.00 | -2.00 | -9.00 | 4.00 | 0.16 | **0.013** | -2.00 | 0.21 | **0.001** |
| CPQ T0 to T2 | 120 | 6.03 | | 12.70 | -51.00 | 47.00 | 4.50 | -1.00 | 12.00 | 0.37 | **<0.001** | 3.00 | 0.37 | **<0.001** |
| CPQ OS T0 | 132 | 5.55 | | 2.97 | 1.00 | 17.00 | 5.00 | 3.00 | 7.00 |  |  | 4.00 |  |  |
| CPQ OS T1 | 119 | 7.13 | | 2.98 | 0.00 | 16.00 | 7.00 | 5.00 | 9.00 |  |  | 6.00 |  |  |
| CPQ OS T2 | 120 | 4.43 | | 2.30 | 0.00 | 10.00 | 4.00 | 3.00 | 6.00 |  |  | 3.00 |  |  |
| OS T0 to T1 | 119 | -1.69 | | 3.03 | -10.00 | 6.00 | -2.00 | -4.00 | 0.00 | 0.35 | **<0.001** | -2.00 | 0.41 | **<0.001** |
| OS T0 to T2 | 120 | 0.97 | | 2.80 | -6.00 | 13.00 | 1.00 | -1.00 | 2.00 | 0.23 | **<0.001** | 1.00 | 0.26 | **<0.001** |
| CPQ FL T0 | 132 | 4.72 | | 3.40 | 0.00 | 15.00 | 4.00 | 2.00 | 7.00 |  |  | 2.00 |  |  |
| CPQ FL T1 | 119 | 6.76 | | 3.93 | 0.00 | 17.00 | 7.00 | 4.00 | 9.00 |  |  | 3.00 |  |  |
| CPQ FL T2 | 120 | 3.16 | | 2.54 | 0.00 | 10.00 | 3.00 | 1.00 | 4.00 |  |  | 1.00 |  |  |
| FL T0 to T1 | 119 | -2.27 | | 4.52 | -14.00 | 15.00 | -2.00 | -5.00 | .00 | 0.34 | **<0.001** | -1.00 | 0.37 | **<0.001** |
| FL T0 to T2 | 120 | 1.44 | | 3.75 | -9.00 | 11.00 | 1.00 | -1.00 | 4.00 | 0.27 | **<0.001** | 1.00 | 0.21 | **<0.001** |
| CPQ EW T0 | 132 | 4.77 | | 5.81 | 0.00 | 31.00 | 2.50 | 0.00 | 7.75 |  |  | 2.00 |  |  |
| CPQ EW T1 | 119 | 3.71 | | 5.12 | 0.00 | 27.00 | 2.00 | 0.00 | 5.00 |  |  | 1.00 |  |  |
| CPQ EW T2 | 120 | 2.48 | | 4.09 | 0.00 | 22.00 | 1.00 | 0.00 | 3.00 |  |  | 1.00 |  |  |
| EW T0 to T1 | 119 | 1.18 | | 5.98 | -20.00 | 29.00 | 0.00 | -1.00 | 4.00 | 0.14 | **0.027** | 0.00 | 0.14 | **0.035** |
| EW T0 to T2 | 120 | 2.29 | | 6.38 | -22.00 | 29.00 | 1.00 | 0.00 | 5.00 | 0.28 | **<0.001** | 1.00 | 0.28 | **<0.001** |
| CPQ SW T0 | 132 | 2.76 | | 3.85 | 0.00 | 20.00 | 1.00 | 0.00 | 3.00 |  |  | 1.00 |  |  |
| CPQ SW T1 | 119 | 2.58 | | 3.51 | 0.00 | 19.00 | 1.00 | 0.00 | 4.00 |  |  | 0.00 |  |  |
| CPQ SW T2 | 120 | 1.47 | | 2.38 | 0.00 | 15.00 | 1.00 | 0.00 | 2.00 |  |  | 0.00 |  |  |
| SW T0 to T1 | 119 | 0.22 | | 4.66 | -17.00 | 20.00 | 0.00 | -2.00 | 1.00 | 0.02 | 0.743 | 0.00 | 0.11 | 0.098 |
| SW T0 to T2 | 120 | 1.33 | | 3.87 | -14.00 | 18.00 | 1.00 | 0.00 | 2.00 | 0.28 | **<0.001** | 0.00 | 0.32 | **<0.001** |
| **Treatment Groups** | | | | | | | | | | | | | | |
| **CB** | | | | | | | | | | | | | | |
| CPQT0 | 70 | 18.51 | | 10.65 | 5.00 | 50.00 | 17.00 | 10.00 | 25.25 |  |  | 9.50 |  |  |
| CPQT1 | 64 | 21.34 | | 12.55 | 6.00 | 59.00 | 18.50 | 11.00 | 28.00 |  |  | 12.00 |  |  |
| CPQT2 | 65 | 11.08 | | 7.27 | 2.00 | 37.00 | 8.00 | 6.00 | 14.00 |  |  | 5.00 |  |  |
| CPQ T0 to T1 | 64 | -3.30 | | 12.14 | -29.00 | 21.00 | -2.50 | -9.75 | 3.75 | 0.18 | **0.045** | -2.00 | 0.23 | **0.009** |
| CPQ T0 to T2 | 65 | 7.25 | | 9.82 | -11.00 | 43.00 | 6.00 | 0.00 | 13.00 | 0.45 | **<0.001** | 4.00 | 0.47 | **<0.001** |
| CPQ OS T0 | 70 | 5.86 | | 2.81 | 1.00 | 15.00 | 5.00 | 4.00 | 7.00 |  |  | 4.00 |  |  |
| CPQ OS T1 | 64 | 7.23 | | 3.02 | 2.00 | 16.00 | 7.00 | 5.00 | 9.75 |  |  | 6.00 |  |  |
| CPQ OS T2 | 65 | 4.15 | | 2.08 | 0.00 | 10.00 | 4.00 | 3.00 | 5.00 |  |  | 3.00 |  |  |
| OS T0 to T1 | 64 | -1.58 | | 2.82 | -10.00 | 5.00 | -1.50 | -3.00 | 0.00 | 0.35 | **<0.001** | -1.00 | 0.40 | **<0.001** |
| OS T0 to T2 | 65 | 1.55 | | 2.60 | -3.00 | 13.00 | 1.00 | 0.00 | 3.00 | 0.41 | **<0.001** | 1.00 | 0.39 | **<0.001** |
| CPQ FL T0 | 70 | 4.93 | | 3.38 | 0.00 | 15.00 | 4.00 | 2.00 | 7.00 |  |  | 2.00 |  |  |
| CPQ FL T1 | 64 | 7.31 | | 3.96 | 0.00 | 17.00 | 7.00 | 4.00 | 10.00 |  |  | 3.00 |  |  |
| CPQ FL T2 | 65 | 3.40 | | 2.65 | 0.00 | 10.00 | 3.00 | 1.50 | 5.00 |  |  | 1.00 |  |  |
| FL T0 to T1 | 64 | -2.50 | | 4.66 | -14.00 | 15.00 | -2.00 | -5.00 | 0.00 | 0.37 | **<0.001** | -2.00 | 0.43 | **<0.001** |
| FL T0 to T2 | 65 | 1.58 | | 3.78 | -8.00 | 11.00 | 2.00 | -1.00 | 4.00 | 0.29 | **<0.001** | 1.00 | 0.21 | **0.017** |
| CPQ EW T0 | 70 | 4.64 | | 4.80 | 0.00 | 20.00 | 3.00 | 1.00 | 8.25 |  |  | 2.00 |  |  |
| CPQ EW T1 | 64 | 4.06 | | 5.73 | 0.00 | 27.00 | 2.00 | 0.00 | 6.00 |  |  | 1.00 |  |  |
| CPQ EW T2 | 65 | 2.02 | | 3.45 | 0.00 | 19.00 | 1.00 | 0.00 | 3.00 |  |  | 0.00 |  |  |
| EW T0 to T1 | 64 | 0.50 | | 5.03 | -14.00 | 15.00 | 1.00 | -2.00 | 3.00 | 0.09 | 0.313 | 1.00 | 0.14 | 0.110 |
| EW T0 to T2 | 65 | 2.58 | | 4.79 | -6.00 | 15.00 | 1.00 | 0.00 | 5.00 | 0.33 | **<0.001** | 1.00 | 0.36 | **<0.001** |
| CPQ SW T0 | 70 | 3.09 | | 3.62 | 0.00 | 15.00 | 2.00 | 1.00 | 4.00 |  |  | 1.00 |  |  |
| CPQ SW T1 | 64 | 2.73 | | 3.74 | 0.00 | 18.00 | 1.00 | 0.00 | 4.00 |  |  | 0.00 |  |  |
| CPQ SW T2 | 65 | 1.51 | | 2.19 | 0.00 | 14.00 | 1.00 | 0.00 | 2.00 |  |  | 0.00 |  |  |
| SW T0 to T1 | 64 | 0.28 | | 4.17 | -13.00 | 13.00 | 0.00 | -2.00 | 1.75 | 0.04 | 0.631 | 0.00 | 0.12 | 0.170 |
| SW T0 to T2 | 65 | 1.52 | | 2.73 | -3.00 | 13.00 | 1.00 | 0.00 | 3.00 | 0.37 | **<0.001** | 1.00 | 0.41 | **<0.001** |
| **PSLB** | | | | | | | | | | | | | | |
| CPQT0 | 62 | 16.97 | | 13.64 | 3.00 | 61.00 | 13.00 | 8.00 | 21.25 |  |  | 7.00 |  |  |
| CPQT1 | 55 | 18.84 | | 10.74 | 3.00 | 58.00 | 16.00 | 10.00 | 27.00 |  |  | 9.00 |  |  |
| CPQT2 | 55 | 12.07 | | 9.59 | 0.00 | 55.00 | 10.00 | 6.00 | 15.00 |  |  | 6.00 |  |  |
| CPQ T0 to T1 | 55 | -1.71 | | 13.84 | -34.00 | 39.00 | -2.00 | -8.00 | 4.00 | 0.14 | 0.143 | -3.00 | 0.18 | 0.054 |
| CPQ T0 to T2 | 55 | 4.60 | | 15.40 | -51.00 | 47.00 | 3.00 | -1.00 | 8.00 | 0.30 | **0.002** | 1.00 | 0.26 | **0.005** |
| CPQ OS T0 | 62 | 5.19 | | 3.12 | 1.00 | 17.00 | 5.00 | 3.00 | 7.00 |  |  | 3.50 |  |  |
| CPQ OS T1 | 55 | 7.00 | | 2.94 | 0.00 | 13.00 | 7.00 | 5.00 | 9.00 |  |  | 6.00 |  |  |
| CPQ OS T2 | 55 | 4.75 | | 2.52 | 0.00 | 10.00 | 4.00 | 3.00 | 7.00 |  |  | 3.00 |  |  |
| OS T0 to T1 | 55 | -1.82 | | 3.28 | -9.00 | 6.00 | -2.00 | -4.00 | 1.00 | 0.35 | **<0.001** | -2.00 | 0.42 | **<0.001** |
| OS T0 to T2 | 55 | 0.27 | | 2.89 | -6.00 | 9.00 | 0.00 | -1.00 | 2.00 | 0.04 | 0.711 | 0.00 | 0.09 | 0.351 |
| CPQ FL T0 | 62 | 4.48 | | 3.42 | 0.00 | 12.00 | 4.00 | 1.00 | 7.00 |  |  | 1.50 |  |  |
| CPQ FL T1 | 55 | 6.13 | | 3.83 | 0.00 | 14.00 | 5.00 | 3.00 | 9.00 |  |  | 2.00 |  |  |
| CPQ FL T2 | 55 | 2.87 | | 2.39 | 0.00 | 9.00 | 2.00 | 1.00 | 4.00 |  |  | 1.00 |  |  |
| FL T0 to T1 | 55 | -2.00 | | 4.38 | -13.00 | 8.00 | -1.00 | -4.00 | 1.00 | 0.29 | **0.002** | -1.00 | 0.29 | **0.002** |
| FL T0 to T2 | 55 | 1.27 | | 3.73 | -9.00 | 10.00 | 1.00 | -1.00 | 4.00 | 0.26 | **0.006** | 0.00 | 0.23 | **0.017** |
| CPQ EW T0 | 62 | 4.90 | | 6.81 | 0.00 | 31.00 | 2.00 | 0.00 | 7.25 |  |  | 1.00 |  |  |
| CPQ EW T1 | 55 | 3.31 | | 4.31 | 0.00 | 24.00 | 2.00 | 1.00 | 5.00 |  |  | 1.00 |  |  |
| CPQ EW T2 | 55 | 3.04 | | 4.71 | 0.00 | 22.00 | 1.00 | 0.00 | 4.00 |  |  | 1.00 |  |  |
| EW T0 to T1 | 55 | 1.96 | | 6.90 | -20.00 | 29.00 | 0.00 | -1.00 | 4.00 | 0.19 | **0.043** | 0.00 | 0.14 | 0.154 |
| EW T0 to T2 | 55 | 1.95 | | 7.89 | -22.00 | 29.00 | 1.00 | 0.00 | 5.00 | 0.21 | **0.026** | 0.00 | 0.19 | **0.048** |
| CPQ SW T0 | 62 | 2.39 | | 4.09 | 0.00 | 20.00 | 1.00 | 0.00 | 2.25 |  |  | 0.00 |  |  |
| CPQ SW T1 | 55 | 2.40 | | 3.24 | 0.00 | 19.00 | 1.00 | 0.00 | 3.00 |  |  | 0.00 |  |  |
| CPQ SW T2 | 55 | 1.42 | | 2.61 | 0.00 | 15.00 | 1.00 | 0.00 | 1.00 |  |  | 0.00 |  |  |
| SW T0 to T1 | 55 | 0.15 | | 5.22 | -17.00 | 20.00 | -1.00 | -2.00 | 1.00 | 0.10 | 0.315 | 0.00 | 0.08 | 0.432 |
| SW T0 to T2 | 55 | 1.11 | | 4.92 | -14.00 | 18.00 | 0.00 | 0.00 | 2.00 | 0.19 | 0.051 | 0.00 | 0.23 | **0.014** |
| **Boy/Girl** | | | | | | | | | | | | | | |
| **BOY** | | | | | | | | | | | | | | |
| CPQT0 | 59 | 15.76 | | 11.50 | 4.00 | 52.00 | 12.00 | 8.00 | 19.00 |  |  | 7.00 |  |  |
| CPQT1 | 54 | 18.76 | | 11.57 | 6.00 | 58.00 | 14.00 | 9.00 | 26.25 |  |  | 8.00 |  |  |
| CPQT2 | 56 | 11.55 | | 9.08 | 0.00 | 55.00 | 8.50 | 7.00 | 12.75 |  |  | 5.00 |  |  |
| CPQ T0 to T1 | 54 | -3.02 | | 11.92 | -27.00 | 28.00 | -2.00 | -10.25 | 3.25 | 0.19 | **0.043** | -2.00 | 0.23 | **0.016** |
| CPQ T0 to T2 | 56 | 4.04 | | 13.47 | -51.00 | 43.00 | 2.50 | -1.00 | 8.00 | 0.33 | **<0.001** | 1.00 | 0.33 | **<0.001** |
| CPQ OS T0 | 59 | 5.56 | | 3.16 | 1.00 | 17.00 | 5.00 | 3.00 | 7.00 |  |  | 4.00 |  |  |
| CPQ OS T1 | 54 | 7.43 | | 3.09 | 2.00 | 16.00 | 7.00 | 5.00 | 10.00 |  |  | 6.00 |  |  |
| CPQ OS T2 | 56 | 4.46 | | 2.50 | 0.00 | 10.00 | 4.00 | 3.00 | 7.00 |  |  | 3.00 |  |  |
| OS T0 to T1 | 54 | -1.81 | | 3.29 | -10.00 | 6.00 | -2.00 | -4.00 | .25 | 0.35 | **<0.001** | -1.50 | 0.38 | **<0.001** |
| OS T0 to T2 | 56 | 1.11 | | 3.18 | -6.00 | 13.00 | 1.00 | .00 | 2.00 | 0.23 | **0.014** | 1.00 | 0.34 | **<0.001** |
| CPQ FL T0 | 59 | 4.93 | | 3.60 | 0.00 | 15.00 | 4.00 | 2.00 | 7.00 |  |  | 1.00 |  |  |
| CPQ FL T1 | 54 | 6.19 | | 4.22 | 0.00 | 17.00 | 5.00 | 3.00 | 9.00 |  |  | 2.00 |  |  |
| CPQ FL T2 | 56 | 3.27 | | 2.53 | 0.00 | 10.00 | 3.00 | 2.00 | 4.00 |  |  | 1.00 |  |  |
| FL T0 to T1 | 54 | -1.43 | | 4.77 | -12.00 | 15.00 | -1.00 | -4.00 | 1.00 | 0.22 | **0.019** | -1.00 | 0.26 | **0.007** |
| FL T0 to T2 | 56 | 1.54 | | 3.75 | -9.00 | 10.00 | 1.00 | -0.75 | 4.00 | 0.30 | **0.001** | 1.00 | 0.25 | **0.008** |
| CPQ EW T0 | 59 | 3.02 | | 4.59 | 0.00 | 21.00 | 1.00 | 0.00 | 4.00 |  |  | 1.00 |  |  |
| CPQ EW T1 | 54 | 2.89 | | 4.64 | 0.00 | 27.00 | 1.00 | 0.00 | 4.25 |  |  | 1.00 |  |  |
| CPQ EW T2 | 56 | 2.14 | | 3.88 | 0.00 | 22.00 | 1.00 | 0.00 | 2.00 |  |  | 1.00 |  |  |
| EW T0 to T1 | 54 | 0.17 | | 5.05 | -14.00 | 15.00 | 0.00 | -2.25 | 2.00 | 0.02 | 0.870 | 0.00 | 0.02 | 0.806 |
| EW T0 to T2 | 56 | 0.82 | | 5.88 | -22.00 | 19.00 | 0.00 | 0.00 | 2.00 | 0.16 | 0.098 | 0.00 | 0.12 | 0.192 |
| CPQ SW T0 | 59 | 2.25 | | 3.37 | 0.00 | 15.00 | 1.00 | 0.00 | 2.00 |  |  | 0.00 |  |  |
| CPQ SW T1 | 54 | 2.26 | | 2.93 | 0.00 | 14.00 | 1.00 | 0.00 | 3.00 |  |  | 0.00 |  |  |
| CPQ SW T2 | 56 | 1.68 | | 3.07 | 0.00 | 15.00 | 1.00 | 0.00 | 2.00 |  |  | 0.00 |  |  |
| SW T0 to T1 | 54 | 0.06 | | 4.17 | -13.00 | 13.00 | 0.00 | -2.00 | 1.00 | 0.06 | 0.516 | 0.00 | 0.08 | 0.425 |
| SW T0 to T2 | 56 | 0.57 | | 3.71 | -14.00 | 13.00 | 1.00 | -0.75 | 1.00 | 0.17 | 0.066 | 0.00 | 0.20 | **0.031** |
| **GIRL** | | | | | | | | | | | | | | |
| CPQT0 | 73 | 19.42 | | 12.44 | 3.00 | 61.00 | 18.00 | 10.00 | 25.00 |  |  | 10.00 |  |  |
| CPQT1 | 65 | 21.37 | | 11.88 | 3.00 | 59.00 | 19.00 | 12.00 | 28.00 |  |  | 12.00 |  |  |
| CPQT2 | 64 | 11.52 | | 7.82 | .00 | 37.00 | 9.00 | 6.00 | 15.75 |  |  | 5.50 |  |  |
| CPQ T0 to T1 | 65 | -2.18 | | 13.77 | -34.00 | 39.00 | -2.00 | -8.50 | 4.50 | 0.13 | **0.130** | -2.00 | 0.19 | **0.030** |
| CPQ T0 to T2 | 64 | 7.78 | | 11.81 | -21.00 | 47.00 | 6.00 | -0.75 | 15.00 | 0.41 | **<0.001** | 5.00 | 0.41 | **<0.001** |
| CPQ OS T0 | 73 | 5.53 | | 2.82 | 1.00 | 15.00 | 5.00 | 3.50 | 7.00 |  |  | 4.00 |  |  |
| CPQ OS T1 | 65 | 6.88 | | 2.88 | 0.00 | 13.00 | 7.00 | 5.00 | 8.00 |  |  | 6.00 |  |  |
| CPQ OS T2 | 64 | 4.39 | | 2.13 | 0.00 | 10.00 | 4.00 | 3.00 | 6.00 |  |  | 3.00 |  |  |
| OS T0 to T1 | 65 | -1.58 | | 2.83 | -8.00 | 5.00 | -2.00 | -4.00 | 0.50 | 0.35 | **<0.001** | -2.00 | 0.43 | **<0.001** |
| OS T0 to T2 | 4 | 0.84 | | 2.44 | -4.00 | 6.00 | 0.50 | -1.00 | 2.00 | 0.24 | **0.006** | 1.00 | 0.21 | **0.019** |
| CPQ FL T0 | 73 | 4.55 | | 3.24 | 0.00 | 14.00 | 4.00 | 2.00 | 7.00 |  |  | 2.00 |  |  |
| CPQ FL T1 | 65 | 7.25 | | 3.64 | 0.00 | 16.00 | 7.00 | 4.00 | 9.00 |  |  | 3.00 |  |  |
| CPQ FL T2 | 64 | 3.06 | | 2.56 | 0.00 | 10.00 | 3.00 | 1.00 | 5.00 |  |  | 0.50 |  |  |
| FL T0 to T1 | 65 | -2.97 | | 4.21 | -14.00 | 5.00 | -2.00 | -5.00 | .00 | 0.43 | **<0.001** | -2.00 | 0.45 | **<0.001** |
| FL T0 to T2 | 64 | 1.36 | | 3.77 | -8.00 | 11.00 | 1.50 | -1.00 | 4.00 | 0.25 | **0.005** | 1.00 | 0.18 | **0.042** |
| CPQ EW T0 | 73 | 6.18 | | 6.32 | 0.00 | 31.00 | 4.00 | 1.00 | 11.00 |  |  | 3.00 |  |  |
| CPQ EW T1 | 65 | 4.40 | | 5.43 | 0.00 | 25.00 | 2.00 | 0.00 | 6.50 |  |  | 2.00 |  |  |
| CPQ EW T2 | 64 | 2.78 | | 4.27 | 0.00 | 19.00 | 1.00 | 0.00 | 3.75 |  |  | 1.00 |  |  |
| EW T0 to T1 | 65 | 2.02 | | 6.58 | -20.00 | 29.00 | 2.00 | -1.00 | 5.00 | 0.25 | **0.003** | 1.00 | 0.24 | **0.005** |
| EW T0 to T2 | 64 | 3.58 | | 6.56 | -15.00 | 29.00 | 3.00 | 0.00 | 7.75 | 0.36 | **<0.001** | 2.00 | 0.40 | **<0.001** |
| CPQ SW T0 | 73 | 3.16 | | 4.17 | 0.00 | 20.00 | 2.00 | 1.00 | 4.00 |  |  | 1.00 |  |  |
| CPQ SW T1 | 65 | 2.85 | | 3.93 | 0.00 | 19.00 | 1.00 | 1.00 | 4.00 |  |  | 1.00 |  |  |
| CPQ SW T2 | 64 | 1.28 | | 1.56 | 0.00 | 7.00 | 1.00 | .00 | 2.00 |  |  | 0.00 |  |  |
| SW T0 to T1 | 65 | 0.35 | | 5.06 | -17.00 | 20.00 | 0.00 | -2.00 | 2.00 | 0.01 | 0.928 | 0.00 | 0.13 | 0.140 |
| SW T0 to T2 | 64 | 2.00 | | 3.92 | -6.00 | 18.00 | 1.00 | 0.00 | 3.00 | 0.37 | **<0.001** | 1.00 | 0.42 | **<0.001** |
| Abbreviations: n, number analysed; SD, standard deviation; Min, lowest registered score; Max, highest registered score; p, p-value; RSF16, Child Perception Questionnaire 16-item Regression Short Form; CPQ, Child Perception Questionnaire; T0, baseline; T1, post alignment; T2, post treatment; OS, oral symptoms; FL, functional limitations; EW, emotional wellbeing; SW, social wellbeing; SF, short form; T0 to T1, score change during alignment phase; T0 to T2, score change during treatment; r, effect size for the Wilcoxon signed-ranks test.  Note: Statistical significance was defined as p<0.05, p-values in bold are statistically significant. | | | | | | | | | | | | | | |
