# Supplementary material for: Oral health-related quality of life, experience and satisfaction in adolescents treated for dental crowding with self-ligating or conventional fixed appliances: a multicentre randomized controlled trial
Source: Eur J Orthod. 2026 Jun 8;48(4):cjag040. doi: 10.1093/ejo/cjag040 (PMC13244279; doi:10.1093/ejo/cjag040)
Supplement: cjag040_Supplementary_Data [file cjag040_supplementary_data.zip › S3. CPQ11-14 score changes_ complete CPQ 11-14 questionnaires.docx]

| **Supplementary Table 3 (S3)**. Sensitivity analysis assessing the impact of imputation using only complete CPQ11-14 and Feldmann’s questionnaires. Within group comparisons were performed using the Wilcoxon signed-ranks test, and between group comparisons using the Mann-Whitney U test. | | | | | | | | |
| --- | --- | --- | --- | --- | --- | --- | --- | --- |
|  |  |  |  |  |  |  | **Wilcoxon signed-ranks test** | |
| Variable | Time | n | Mean | SD | n | Mean change | r | p-value |
| CPQ T0 to T1 | T0 | 111 | 13.94 | 11.04 | 88 | -2.78 | 0.21 | **0.005** |
|  | T1 | 104 | 17.04 | 11.44 |  |  |  |  |
| OS T0 to T1 | T0 | 123 | 4.44 | 2.60 | 88 | -1.75 | 0.38 | **<0.001** |
|  | T1 | 114 | 6.21 | 2.74 |  |  |  |  |
| FL T0 to T1 | T0 | 129 | 2.67 | 2.77 | 88 | -2.19 | 0.38 | **<0.001** |
|  | T1 | 111 | 4.70 | 3.47 |  |  |  |  |
| EW T0 to T1 | T0 | 123 | 4.88 | 5.95 | 88 | 1.33 | 0.15 | **0.027** |
|  | T1 | 114 | 3.79 | 5.18 |  |  |  |  |
| SW T0 to T1 | T0 | 120 | 2.77 | 3.94 | 88 | 0.29 | 0.02 | 0.772 |
|  | T1 | 113 | 2.36 | 3.14 |  |  |  |  |
| CPQ T0 to T2 | T0 | 111 | 13.94 | 11.04 | 95 | 5.83 | 0.37 | **<0.001** |
|  | T2 | 110 | 8.56 | 6.87 |  |  |  |  |
| OS T0 to T2 | T0 | 123 | 4.44 | 2.60 | 95 | 0.96 | 0.24 | **<0.001** |
|  | T2 | 116 | 3.48 | 2.08 |  |  |  |  |
| FL T0 to T2 | T0 | 129 | 2.67 | 2.77 | 95 | 1.06 | 0.26 | **<0.001** |
|  | T2 | 115 | 1.46 | 1.82 |  |  |  |  |
| EW T0 to T2 | T0 | 123 | 4.88 | 5.95 | 95 | 2.38 | 0.28 | **<0.001** |
|  | T2 | 118 | 2.53 | 4.11 |  |  |  |  |
| SW T0 to T2 | T0 | 120 | 2.77 | 3.94 | 95 | 1.29 | 0.27 | **<0.001** |
|  | T2 | 117 | 1.48 | 2.39 |  |  |  |  |
|  |  |  |  |  |  | **Mann-Whitney U test** | | |
| Variable | Treatment group | n | Mean change | SD | r | p-value | | |
| CPQ T0 to T1 | CB | 54 | -3.63 | 11.49 | 0.01 | 0.927 | | |
|  | PSLB | 34 | -1.44 | 12.24 |  |  |  |  |
| OS T0 to T1 | CB | 59 | -1.90 | 2.63 | 0.02 | 0.860 | | |
|  | PSLB | 47 | -1.55 | 3.08 |  |  |  |  |
| FL T0 to T1 | CB | 61 | -2.39 | 4.04 | 0.09 | 0.356 | | |
|  | PSLB | 47 | -1.91 | 3.58 |  |  |  |  |
| EW T0 to T1 | CB | 59 | 0.54 | 5.11 | 0.04 | 0.662 | | |
|  | PSLB | 48 | 2.29 | 6.37 |  |  |  |  |
| SW T0 to T1 | CB | 61 | 0.61 | 3.87 | 0.20 | **0.044** | | |
|  | PSLB | 43 | -0.16 | 3.90 |  |  |  |  |
| CPQ T0 to T2 | CB | 58 | 6.53 | 9.36 | 0.14 | 0.187 | | |
|  | PSLB | 37 | 4.73 | 13.71 |  |  |  |  |
| OS T0 to T2 | CB | 62 | 1.31 | 2.24 | 0.21 | **0.031** | | |
|  | PSLB | 45 | 0.49 | 2.71 |  |  |  |  |
| FL T0 to T2 | CB | 64 | 1.14 | 2.96 | 0.05 | 0.624 | | |
|  | PSLB | 49 | 0.96 | 2.79 |  |  |  |  |
| EW T0 to T2 | CB | 62 | 2.56 | 4.84 | 0.03 | 0.751 | | |
|  | PSLB | 49 | 2.14 | 8.20 |  |  |  |  |
| SW T0 to T2 | CB | 61 | 1.62 | 2.78 | 0.17 | 0.075 | | |
|  | PSLB | 48 | 0.88 | 5.08 |  |  |  |  |
| Variable | Sex | n | Mean | SD | r | p-value | | |
| CPQ T0 to T1 | BOY | 37 | -3.19 | 10.70 | 0.06 | 0.588 | | |
|  | GIRL | 51 | -2.49 | 12.58 |  |  |  |  |
| OS T0 to T1 | BOY | 47 | -1.77 | 2.91 | 0.01 | 0.950 | | |
|  | GIRL | 59 | -1.73 | 2.79 |  |  |  |  |
| FL T0 to T1 | BOY | 49 | -1.22 | 3.85 | 0.21 | 0.030 | | |
|  | GIRL | 59 | -2.98 | 3.67 |  |  |  |  |
| EW T0 to T1 | BOY | 48 | 0.31 | 5.20 | 0.20 | **0.038** | | |
|  | GIRL | 59 | 2.15 | 6.07 |  |  |  |  |
| SW T0 to T1 | BOY | 44 | 0.18 | 3.79 | 0.07 | 0.497 | | |
|  | GIRL | 60 | 0.37 | 3.99 |  |  |  |  |
| CPQ T0 to T2 | BOY | 44 | 4.07 | 10.99 | 0.18 | 0.073 | | |
|  | GIRL | 51 | 7.35 | 11.30 |  |  |  |  |
| OS T0 to T2 | BOY | 50 | 1.10 | 2.88 | 0.02 | 0.874 | | |
|  | GIRL | 57 | 0.84 | 2.07 |  |  |  |  |
| FL T0 to T2 | BOY | 55 | 1.18 | 2.82 | 0.04 | 0.675 | | |
|  | GIRL | 58 | 0.95 | 2.95 |  |  |  |  |
| EW T0 to T2 | BOY | 51 | 0.82 | 6.11 | 0.22 | **0.018** | | |
|  | GIRL | 60 | 3.70 | 6.60 |  |  |  |  |
| SW T0 to T2 | BOY | 49 | 0.35 | 3.68 | 0.20 | **0.038** | | |
|  | GIRL | 60 | 2.07 | 4.04 |  |  |  |  |
| Feldmann’s questionnaire item number | Treatment group | n | Mean | SD | r | p-value | | |
| 1 | CB | 61 | 9.77 | 0.59 | 0.15 | 0.118 | | |
|  | PSLB | 56 | 9.57 | 0.83 |  |  |  |  |
| 2 | CB | 61 | 9.61 | 0.94 | 0.14 | 0.121 | | |
|  | PSLB | 56 | 9.45 | 0.95 |  |  |  |  |
| 3 | CB | 61 | 9.82 | 0.43 | 0.25 | **0.007** | | |
|  | PSLB | 56 | 9.45 | 0.89 |  |  |  |  |
| 4 | CB | 61 | 9.62 | 0.69 | 0.20 | **0.033** | | |
|  | PSLB | 56 | 9.20 | 1.21 |  |  |  |  |
| 5 | CB | 61 | 1.61 | 1.41 | 0.04 | 0.652 | | |
|  | PSLB | 55 | 1.67 | 1.41 |  |  |  |  |
| 6 | CB | 61 | 6.51 | 4.08 | 0.05 | 0.600 | | |
|  | PSLB | 56 | 6.79 | 3.97 |  |  |  |  |
| 7 | CB | 61 | 8.64 | 1.74 | 0.08 | 0.418 | | |
|  | PSLB | 56 | 8.91 | 1.52 |  |  |  |  |
| 8 | CB | 60 | 9.30 | 1.00 | 0.01 | 0.938 | | |
|  | PSLB | 53 | 9.25 | 1.11 |  |  |  |  |
| 9 | CB | 59 | 9.68 | 0.57 | 0.12 | 0.228 | | |
|  | PSLB | 51 | 9.80 | 0.45 |  |  |  |  |
| 10 | CB | 58 | 9.66 | 0.76 | 0.04 | 0.673 | | |
|  | PSLB | 52 | 9.79 | 0.46 |  |  |  |  |
| 11 | CB | 60 | 8.18 | 2.10 | 0.09 | 0.341 | | |
|  | PSLB | 53 | 7.96 | 2.16 |  |  |  |  |
| 12 | CB | 60 | 5.88 | 2.64 | 0.15 | 0.116 | | |
|  | PSLB | 53 | 5.08 | 2.55 |  |  |  |  |
| 13 | CB | 60 | 5.92 | 2.38 | 0.09 | 0.315 | | |
|  | PSLB | 53 | 5.47 | 2.22 |  |  |  |  |
| 14 | CB | 61 | 3.03 | 2.18 | 0.01 | 0.937 | | |
|  | PSLB | 54 | 3.06 | 2.17 |  |  |  |  |
| 15 | CB | 61 | 2.16 | 1.93 | 0.02 | 0.806 | | |
|  | PSLB | 54 | 2.30 | 2.11 |  |  |  |  |
| 16 | CB | 60 | 2.98 | 2.44 | 0.05 | 0.626 | | |
|  | PSLB | 53 | 3.11 | 2.60 |  |  |  |  |
| 17 | CB | 61 | 1.31 | 0.96 | 0.03 | 0.627 | | |
|  | PSLB | 54 | 1.24 | 0.99 |  |  |  |  |
| 18 | CB | 60 | 4.27 | 2.38 | 0.06 | 0.552 | | |
|  | PSLB | 53 | 4.04 | 2.44 |  |  |  |  |
| Abbreviations: CPQ, Child perception questionnaire (11-14); n, number analysed; SD, standard deviation; T0, baseline; T1, post alignment; T2, post treatment; OS, oral symptoms; FL, functional limitations; EW, emotional wellbeing; SW, social wellbeing; CB, conventional bracket group; PSLB, passive self-ligating bracket group; r, effect size for between group comparison using Mann-Whitney U test.  Note: Items 6, 9, 34 in the CPQ11-14 were excluded, and sensitivity analysis based on complete questionnaires were performed. This analysis indicate that imputation had only a limited effect on the effect sizes and p‑values.  Statistical significance was defined as p<0.05, statistically significant p-values are shown in bold are. | | | | | | | | |
